# Coping with global warming: Adult thermal thresholds in four pestiferous *Anastrepha* species determined under experimental laboratory conditions and development/survival times of immatures and adults under natural field conditions

**DOI:** 10.3389/fphys.2022.991923

**Published:** 2022-10-11

**Authors:** Larissa Guillén, Carlos Pascacio-Villafán, Ixchel Osorio-Paz, Rafael Ortega-Casas, Erick Enciso-Ortíz, Alma Altúzar-Molina, Olinda Velázquez, Martín Aluja

**Affiliations:** Red de Manejo Biorracional de Plagas y Vectores, Clúster Científico y Tecnológico BioMimic®, Instituto de Ecología, A. C. —INECOL, Xalapa, Veracruz, Mexico

**Keywords:** global warming, thermal thresholds, life-history traits, physiology, *Anastrepha*, Diptera: Tephritidae

## Abstract

Climate change, particularly global warming, is disturbing biological processes in unexpected ways and forcing us to re-study/reanalyze the effects of varying temperatures, among them extreme ones, on insect functional traits such as lifespan and fecundity/fertility. Here we experimentally tested, under both laboratory and field conditions, the effects of an extreme range of temperatures (5, 10, 15, 20, 30, 40, and 45 °C, and the naturally varying conditions experienced in the field), on survivorship/lifespan, fecundity, and fertility of four pestiferous fruit fly species exhibiting contrasting life histories and belonging to two phylogenetic groups within the genus *Anastrepha*: *A. ludens, A. obliqua, A. striata*, and *A. serpentina*. In the field, we also measured the length of the entire life cycle (egg to adult), and in one species (*A. ludens*), the effect on the latter of the host plant (mango and grapefruit). Under laboratory conditions, none of the adults, independent of species, could survive a single day when exposed to a constant temperature of 45 °C, but *A. striata* and *A. serpentina* females/males survived at the highly contrasting temperatures of 5 and 40 °C at least 7 days. Maximum longevity was achieved in all species at 15 °C (375, 225, 175 and 160 days in *A. ludens, A. serpentina, A. striata* and *A. obliqua* females, respectively). *Anastrepha ludens* layed many eggs until late in life (368 days) at 15 °C, but none eclosed. Eclosion was only observed in all species at 20 and 30 °C. Under natural conditions, flies lived ca. 100 days less than in the laboratory at 15 °C, likely due to the physiological cost of dealing with the highly varying environmental patterns over 24 h (minimum and maximum temperatures and relative humidity of ca. 10–40 °C, and 22–100%, respectively). In the case of *A. ludens*, the immature’s developmental time was shorter in mango, but adult survival was longer than in grapefruit. We discuss our results considering the physiological processes regulating the traits measured and tie them to the increasing problem of global warming and its hidden effects on the physiology of insects, as well as the ecological and pest management implications.

## Introduction

The effects of climate change such as global warming and environmental moisture changes are disturbing biological cycles in unexpected ways and forcing us to re-study/reanalyze processes that were well understood in the past (Wang and Schimel, 2003; Parmesan, 2006; Jaworski and Hilszczański, 2013). As temperature and humidity lie at the heart of basically all biological processes, it comes as no surprise that global warming is reaping havoc in both natural and anthropic environments, among them agroecosystems, modifying for example rainfall patterns and relative humidity (Trenberth, 2011; Jiang et al., 2015; Byrne and O’Gorman, 2016) or temperature cycles in such a way that cold/warm spells can be experienced in the middle of summer or winter (Yan et al., 2002; Ratajczak, 2020; Yu et al., 2022). This in turn influences tree flowering/fruit ripening patterns and can modify relationships with pests, which also suffer alterations in their biological cycles or biological attributes such as fecundity, egg survival, longevity, and duration of immature cycles (Jaworski and Hilszczański, 2013; Carnicer et al., 2018; Huang et al., 2020). The effects can be apparent (e.g., decrease in body size (Carnicer et al., 2018), increase in the number of generations per year (Lisbôa et al., 2020; Rashmi et al., 2020)) or subtle/hidden (e.g., slight modifications in hydrocarbon structures in the *epidermis* altering recognition between mating partners (Chung and Carroll, 2015; Sentis et al., 2015; Boullis et al., 2016; Menzel et al., 2018) or alterations in the interactions of odorant/pheromone binding proteins (Weng et al., 2015; Boullis et al., 2016).

Insects are adapted to live in wide temperature ranges, all the way from subzero to extremely hot temperatures, although each species has an optimal range of temperatures where their fitness is maximized (Chapman, 1998). Thermal and humidity stress are two of the most important barriers to the development and survival of insects (Chang et al., 2008; Richter et al., 2010). Since temperature gradients in the field can affect the physiology, reproduction, and distributions of insects, many of them with significant agroecological importance, research on their thermal thresholds remains a relevant scientific endeavor.

Among the most notorious pests worldwide, fruit flies (Diptera: Tephritidae) stand out because of their direct damage to fruit (larvae feed in the pulp rendering the fruit unmarketable) and the quarantine restrictions their presence trigger (Aluja and Mangan, 2008; Qin et al., 2015). Given their economic impact, studies on thermal thresholds and direct effect of temperature on fruit fly biology and behavior have been numerous and started at the beginning of last century. The pioneering studies by Darby and Kapp (1933), McPhail and Bliss (1933), Meats (1976), Meats (1984), Meats (1989), among others stand out in this respect. Other studies on temperature relationships on various biological attributes have been performed with the Mediterranean fruit fly, *Ceratitis capitata* Wiedemann (Duyck and Quilici, 2002; Nyamukondiwa and Terblanche, 2009), the Olive fly, *Bactrocera oleae* Gmelin (Genç and Nation, 2008; Wang et al., 2012), the Oriental fruit fly, *Bactrocera dorsalis* (Hendel) (Samayoa et al., 2018; Motswagole et al., 2019; Rashmi et al., 2020), the Apple Maggot fly, *Rhagoletis pomonella* (Walsh) (Reissig et al., 1979; Kasana and AliNiazee, 1994; Drummond and Collins, 2019) and various other species (Liu and Ye, 2009; Adly, 2016; Rull et al., 2016; Bayoumy et al., 2021). Broadly speaking, temperature affects the reproduction and development of tephritid flies (Telles-Romero et al., 2011; Bolzan et al., 2017; Fiaboe et al., 2021), mainly by influencing the metabolic processes that are linked to their development (Moloń et al., 2020).

In the case of flies within the genus *Anastrepha*, most work on temperature relationships stems from the United States, Mexico, and Brazil. Variation of developmental time at different temperature conditions have been studied in the Mexican fruit fly *Anastrepha ludens* (Loew) (Darby and Kapp, 1933; McPhail and Bliss, 1933; Flitters and Messenger, 1965; Leyva-Vazquez, 1988; Thomas, 1997; Hallman et al., 2005; Aluja et al., 2010) and the Sapote fruit fly, *Anastrepha serpentina* Wiedemann (Shaw and Starr, 1946). Thomas (1997) determined that larvae of *A. ludens* survive inside fruit when temperatures drop below zero. Other studies have been performed on *Anastrepha suspensa* (Loew) (Sivinski et al., 2007) and *Anastrepha grandis* (Macquart) (Bolzan et al., 2017; Silva et al., 2019; Lisbôa et al., 2020; Teixeira et al., 2021; Teixeira et al., 2022).

Based on the need to retake this topic because of the phenomenon of Global Warming and its possible effect on the management of pestiferous species within *Anastrepha*, here we report the results of a broad study aimed at determining the effect of temperature on basic biological parameters such as duration of the life cycle, egg and adult survival, female fecundity/egg fertility and adult longevity of wild *A. ludens*, *Anastrepha obliqua* (Macquart), *A. serpentina* and *Anastrepha striata* (Schiner). The four species are distributed in the tropical zones of Mexico where temperatures tend to be warm, but in the case of *A. ludens* it has been recently expanding its distribution range invading high altitude, temperate areas where apples (*Malus* × *domestica* Borkh.) and pears (*Pyrus communis* L.) are grown (M. Aluja personal observations; Ruiz-Montoya et al., 2020). We note that these species belong to two different species groups which render our study more robust and interesting. *Anastrepha ludens* and *A*. *obliqua* belong to the *fraterculus* group (Smith-Caldas et al., 2001; Mengual et al., 2017), while *A*. *serpentina* and *A*. *striata* to the *serpentina* group (Norrbom, 2002), although Mengual et al. (2017) placed them again in separate groups. Studies were conducted under natural conditions (under a tree in an orchard) and in bioclimatic chambers. As we wanted to test extreme as well as “typical” temperatures to determine the adaptation potential of these flies to the anomalous worldwide temperature patterns, we experimented with the following temperature regimes: 5, 10, 15, 20, 30, 40, and 45°C. Based on what has been published on temperature effects on fruit flies (reviewed above), we predicted that within the *fraterculus* species group, adults of *A. obliqua*, a species commonly found in very hot, drier tropical areas (temperatures can easily reach 40°C or over at midday), would tolerate high temperatures better than species such as *A. ludens*. We also predicted that within the *serpentina* species group, *A*. *serpentina* would better tolerate extremely high temperatures. We further predicted that flies exposed to the highly variable field conditions, would exhibit shorter lifespans than flies exposed to similarly averaged temperatures but devoid of variance. Finally, we predicted that *A*. *ludens* would suffer the most under extremely hot temperatures, with the end results that the distribution of this species would shift towards higher altitude biomes. Our findings are discussed in the light of the physiological processes likely modulating the responses observed.

## Materials and methods

### Insect collection and maintenance

In the case of both laboratory and field studies (details follow), the four fly species used in the experiments, *A. ludens*, *A. obliqua*, *A. striata,* and *A. serpentina*, stemmed from a semi-wild colony kept under laboratory conditions. By semi-wild we mean first-fourth generation adults that originated from wild individuals that were reared in their natural hosts (grapefruit [*Citrus* x *paradisi* Macfad], mango [*Mangifera indica* L.], guava [*Psidium guajava* L.], and sapodilla [*Manilkara sapota* (L.) P. Royen], respectively, for *A. ludens*, *A. obliqua*, *A. striata* and *A. serpentina*), at laboratory environmental conditions (26 ± 1°C, 60 ± 5% RH and 12:12 h L: D photoperiod). Fruit was placed over plastic baskets with many openings over 27 × 13 × 39 (length, width, depth) cm plastic washbowls with vermiculite as a rearing medium on the floor. Third-stage larvae jumped out of the rotten fruit to pupate. Subsequently, the pupae were collected and placed in 100 ml plastic containers with vermiculite, which was sprinkled with a 0.2% sodium benzoate solution every third day until emergence to prevent the development of pathogens, particularly bacteria and fungi. Once the first flies started to emerge, the container with pupae was transferred to 30 × 30 × 30 cm Plexiglas cages with ample aeration (walls and roof were covered with fiberglass mesh) kept at ambient conditions (27 ± 1°C, 65 ± 5% RH, and light: dark 12:12 h). Inside cages, newly emerged flies were provided with *ad libitum* food (a mixture of sugar: hydrolyzed yeast protein 3:1) and water until they were transferred to the experimental units for field or laboratory experiments (details follow).

### Experiment 1—Laboratory conditions

Adults of the four species were exposed to seven temperature treatments (5, 10, 15, 20, 30, 40, and 45°C). For each fly species (i.e., *A. ludens*, *A. obliqua*, *A. striata* and *A. serpentina*), temperature treatments were tested in two parts. First, four Lumistell growth chambers (Model ICP-20) were programmed with temperatures of 5, 10, 15, and 20°C. Each chamber housed five 20 × 20 × 20 cm Plexiglas cages with plastic mesh walls and 20 newly emerged flies (10 females and 10 males) of one species. The cage with flies was considered as the replication unit. When all the flies in all five cages died, chambers were cleaned and scheduled for the remaining temperature treatments (30, 40, and 45°C). As the presence of the different fly species is tied to the availability of their host plants along the year, we ran the tests in blocks of species. That is, at any given moment, only one species was studied, then the next and so on, until all species were covered. But given that we worked with tightly controlled temperature and relative humidity conditions, this did not preclude us from comparing the results among species. The varying temperatures were kept constant throughout the experiment (day one until the last fly died). For all treatments, photoperiod and relative humidity inside chambers were, respectively, 12:12 h (L: D) and 65 ± 5%. Inside cages, we hung daily one colored, 3 cm diam agar sphere wrapped in Parafilm as oviposition substrates to measure female’s fecundity. The color of the spheres was green (910 μL of foliage-green, food grade dye Colorchef in 900 mL of water and 29.4 g of agar) in the case of *A. ludens*, *A. obliqua* and *A. striata*, and light brown (150 µL of yellow dye [yellow 370L, Deimp], 50 µL of red dye [red 370L, Deimp] and 25 µL of black dye [black 370L, Deimp] in 900 ml of water and 29.4 g of agar) for *A. serpentina* (as their common hosts such as *M. sapota* or *Pouteria sapota* (Jacq.) Moore & Stearn have a light brown skin). Five replicates (i.e., one cage per replicate) were run for each temperature and fly species. Every day, adult survival, fecundity, and egg fertility were measured. Eggs were dissected daily from the agar spheres and placed over a moist blue polysilk cloth in turn placed inside a 9 cm covered Petri dish to measure fecundity (number of eggs) and fertility (proportion of egg hatched). The explanatory variables of the experimental design were: i) the temperature measured in a continuous scale from 5 to 45°C, and ii) the fly species as a categorical variable with four levels: *A. ludens*, *A. obliqua*, *A. striata*, and *A. serpentina*. The response variables were: i) the life expectancy of adult females and males (mean time in days from adult emergence until death), ii) the estimated daily egg production per female, iii) egg hatch (proportion) and iv) egg development time (in days).

### Experiment 2—Natural field conditions

**FIGURE 1:**
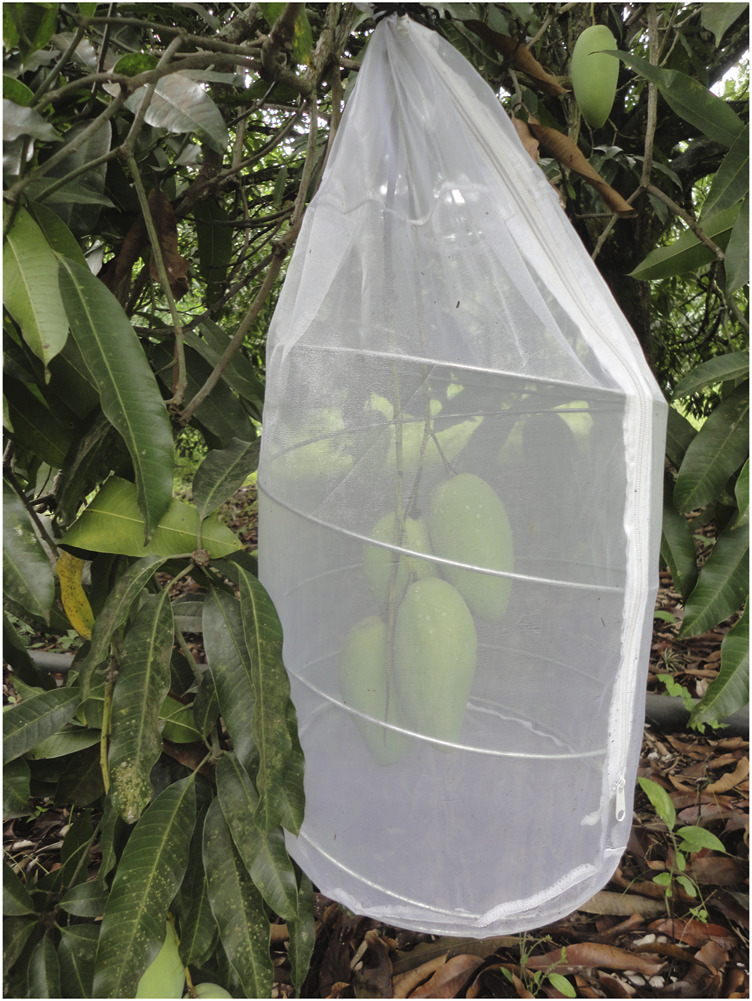
White organza fabric cage with mango fruit cv ‘Manila’ exposed to gravid females (three females per fruit) during 24 h in the field experiment to generate fruit infestation.

As conditions in the field are not stable and considerable fluctuations occur along the 24 h of a day (particularly between mid-day and early morning hours), we also determined the length of the life cycle and adult life span, under natural field conditions, and measured every 15 min temperature and relative humidity throughout the experiment in the studies sites using a Datta logger Hobo Pro v2. As was the case with the laboratory studies, fly availability depended on host fruiting phenology and that is the reason why the observations per species could not be carried out simultaneously. We started by allowing sexually mature females to lay eggs into their respective hosts in fruit naturally attached to bagged tree branches. We worked in the following localities in Veracruz, Mexico (I) Apazapan (19°19′18″N, 96°43′02″W) with *A. ludens* infesting ‘Manila’ mango from May 2014 to March 2015, *A. striata* infesting guava from November 2017 to August 2018, and *A. serpentina* sapodilla from May 2017 to November 2017; (II) Tolome Paso de Ovejas (19°16′00″N, 96°22′54″W) with *A. ludens* infesting ‘Marsh’ grapefruit from December 2014 to September 2015 and (III) Ídolos, Actopan (19°24′44″N, 96°31′15″W) with *A. obliqua* infesting ‘Manila’ mango from June 2015 to February 2016. For all types of fruit and locations, we used the following procedure: in ten trees, we randomly selected branches containing five healthy fruits. Five weeks before running tests, the branches were covered with gauze bags to protect the fruit from the oviposition activity of wild flies or the attack by other insects (e.g., beetles, wasps, caterpillars). When the fruit were close to reaching maturity, the protective bag was removed from each branch and replaced with a white organza fabric cage (Figure 1). Fifteen, 15-d old, sexually mature, mated female flies of each species, respectively, were released into each cage and provided with food and water *ad libitum* (same food as described above). Flies were allowed to lay eggs over a 24-h period, after which time they were removed. All bagged fruit were left until the first fruit was naturally abscised from the branch at which moment, the rest of the fruits were harvested and removed from the cage and, depending on their size, placed in individual plastic 500- or 1000-ml containers with aerated lids and vermiculite in the bottom to allow for larval pupation. We kept one fruit per container. The containers with fruits were arranged on shelves inside a field cage placed under the canopy of one of the trees from which the infested fruit stemmed to expose them to natural ambient environmental conditions throughout the study period. The date on which the third-stage larvae naturally abandoned the fruit, the pupation day, adult emergence dates and sex were recorded daily. The day of emergence, three male and three female flies were isolated in new plastic cages with artificial diet and water *ad libitum* to evaluate lifespan, fertility, fecundity, and length of the complete life cycle, also under completely natural conditions (4–7 replicates per treatment).

The following parameters were measured in both experiments:

**Length of life cycle:** Time elapsed between egg deposition and adult emergence and new egg deposition (sexual maturity of adults).

**Lifespan.** To determine median and maximal survival, the number of dead flies was scored daily in each cage since the first day of life.

**Fecundity and Fertility.** To estimate fecundity and fertility, starting at day eight (when the first eggs were recorded on cage walls), flies inside cages were offered an agar sphere into which they could oviposit. The spheres were dissected daily to retrieve the eggs, which were incubated in Petri dishes containing a blue polysilk cloth on top of cotton moistened with 0.2% sodium benzoate. In Experiment 1, the Petri dishes were placed inside the bioclimatic chambers and exposed to the same temperature regimes the mothers and fathers were exposed to. In the case of Experiment 2 (field), the eggs were kept at ambient conditions. The number of eggs laid per female (fecundity) and egg hatch (fertility) were recorded daily. We note that due to the complexities experienced in the field, among them personnel safety, we only recorded fecundity and egg hatch for a period of 10 continuous days under field conditions. Under laboratory conditions, measurements were made daily until the last egg was oviposited into the agar spheres.

**Length of complete life cycle.** To assess the effect temperature (environmental conditions) on the length of the life cycle, eggs from the first 10 Petri dishes were transferred to a standard artificial larval diet (details in Pascacio-Villafán et al., 2016), and the number of larvae developing as well as the time it took them to pupate were recorded. The pupae obtained were isolated in new containers with moistened vermiculite. In Experiment 1, they were placed in the bioclimatic chambers with the temperature from which the larvae and parents came from. In Experiment 2 (field) the pupae were maintained at ambient conditions. The date of emergence of each individual and the sex were recorded and after this, they were monitored daily until death.

### Statistical analyses

Data on flies’ survival (proportion) from laboratory experiments were first analyzed with “Kaplan-Meier” survival curves using the software Graph Pad Prism 6 (CA, United States). Curves were constructed to examine the time at which 50% of the fly populations perished. First, curves were constructed to analyze differences in the survival of the different fly species at each of the temperatures tested and differences between females and males by fly species at each temperature. Then, the comparison focused on differences in temperatures for each fly species. The differences among the means of the median lifespans of flies were analyzed with Kruskal–Wallis and U-Mann-Whitney post hoc tests (Brooks et al., 1994). The differences between the survival means between females and males per species and temperature were analyzed with a *t*-Test for Dependent Samples (i.e., females and males were not independent from one another as they were grouped together in each replicated cage) with Statistica 10® software (Stat Soft, Inc., 2011).

Data on mean female and male life expectancy (days), daily egg production per female (no. of eggs), egg hatch (proportion) and egg development time (days) of flies from the laboratory trials were analyzed using response surface methods (Anderson and Whitcomb, 2005). The goal was to model the response variables as a function of main and interaction effects of the explanatory variables (temperature in a continuous scale and fly species as a categorical variable). Polynomial models were fitted to the values of each response variable and analyzed by analysis of variance (ANOVA). Analyses were performed following the procedure of the Design-Expert® 10 software (Anderson and Whitcomb, 2005). To improve the normal distribution and constant variance of the residuals of models, data on female and male life expectancy were natural log-transformed, data on the estimated number of eggs per female per day were natural log-transformed after adding a constant value of 0.051 to the data, and data on egg hatch were logit transformed. Transformations were based on Box-Cox analyses and the graphical examination of the model residuals (Anderson and Whitcomb, 2005).

We used descriptive statistics to show the variability of temperatures and relative humidity during the field experiments at each site. Kaplan-Meier survival curves were also constructed to examine the survival of flies in the field. To compare the means of median survival between species (considering females and males together), we used the same method described before for the laboratory experiments (i.e., the Kruskal–Wallis and U-Mann-Whitney post hoc tests). To determine if the survival of *A. ludens* was affected by the type of host fruit as well as male and female differences in all species, survival curves were analyzed using Log-rank (Mantel-Cox) tests run with the Graph Pad Prism 6 (CA, United States). Mean fly survival in the field for all species was analyzed by means of one-way ANOVAs.

Data on the development time of the immature stages of the different fly species under field conditions were analyzed with a non-parametric Kruskal–Wallis test, as the residuals of linear models fitted to the data did not comply with the assumptions of normality and homoscedasticity. Finally, for each immature stage, we performed multiple pairwise comparisons of the median values of fly species with the Dwas-Steel-Critchlow-Fligner test (Hollander and Wolfe, 1999). Analyses were performed with XLSTAT.

**Hypothetical scenario of *A. ludens* invasion to two places in Europe.** After models were fitted to experimental data, a prediction analysis (Anderson and Whitcomb, 2005) was used to formulate scenarios of the potential invasion and establishment of *A. ludens* in temperate areas under global warming conditions (Birke et al., 2013; Aluja et al., 2014). We focused on Switzerland as a scenario because it is one of the European countries that has suffered the most from the ravages of climate change and associated insect pest problems (Schneider et al., 2021), including the recent invasion of the American tephritid fruit fly *Rhagoletis completa* Cresson (Aluja et al., 2011). We retrieved the 2021 monthly average temperature data in Basel (Latitude: 47.5584°N, Longitude: 7.5733°E) and Geneva (Latitude: 46.2042°N, Longitude: 6.1431°E), Switzerland, from the POWER Project CERES/MERRA2 Native Resolution Monthly and Annual on 2022/07/01, National Aeronautics and Space Administration (NASA) Langley Research Center (LaRC) Prediction of Worldwide Energy Resource (POWER) Project funded through the NASA Earth Science/Applied Science Program. The reason for choosing these two sites in Switzerland was based on having represented two regions (Basel in the northwestern and Geneva in the southwest) in which apples (*M. × domestica*) and pears (*P. communis*) are produced (Bravin, 2013). Apples and pears are naturally infested by *A. ludens* in Mexico (M.A. personal observation; Norrbom, 2004). We estimated the spring (April-June), summer (July-September), fall (October-December) and winter (January-March) mean temperatures of each site and considered a 1.3°C increase in temperature for the next 100 years based on Begert et al. (2019). Then, based on the fitted models, we predicted the mean life expectancy of females, the number of eggs per female per day and egg hatch for each of the hypothetical temperatures considered in the 100-year period projection. We coupled this information with the natural history theory and physiological understanding of *A. ludens* to surmise whether *A. ludens* could invade and establish in Switzerland.

## Results

### Kaplan-Meier survival curves of four *Anastrepha* spp. under laboratory conditions

**FIGURE 2:**
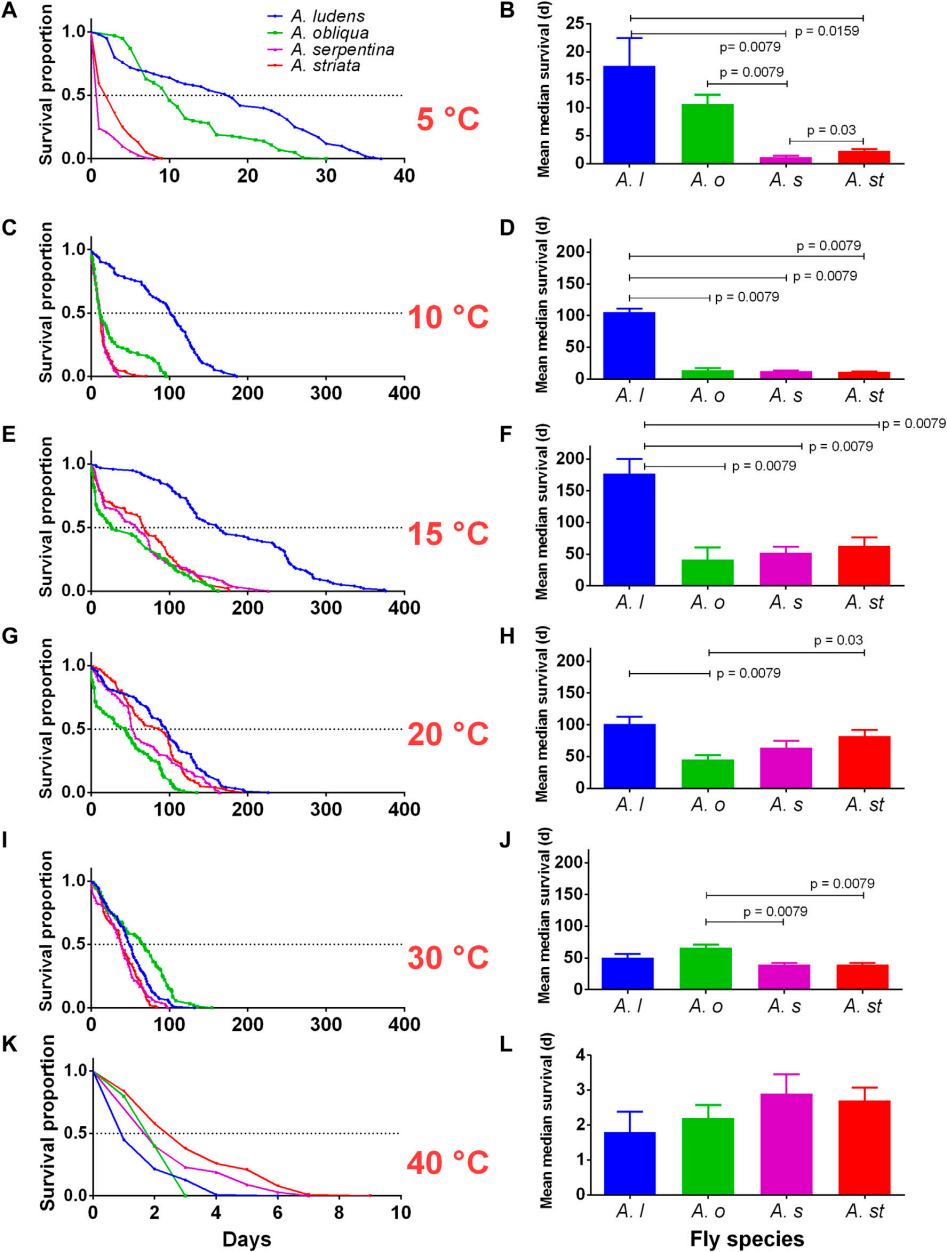
Adult survival (days) of four *Anastrepha* species (*A. ludens, A. obliqua, A. serpentina,* and *A. striata*) exposed to different temperatures (5, 10, 15, 20, 30,40°C) in bioclimatic chambers. **(A,C,E,G,I,K)** Kaplan-Meier survival curves by temperature; the dotted horizontal line represents the mean of the medians of all replicates. **(B,D,F,H,J,L)** Means ± SE of median survival (days) of five replicates (cage with flies) per species. Horizontal bars represent pairwise comparations with Mann-Whitney tests when clear differences were noticed. We left the data for 45°C out as in all species no adult survived more than 24 h.

We found clear statistical differences in the survival of the four fly species studied at the different temperatures tested (Table 1; Figure 2). At 5°C, *A. ludens* and *A. obliqua* had a longer lifespan than *A. serpentina* and *A. striata* (Figures 2A,B). While *A. ludens* at 10 and 15°C had a longer lifespan than the other three fly species (Figures 2C–F), with a mean (±SE) maximal survival of 340 ± 17.62 days at 15°C (Figure 2E; Table 1). All fly species reached maximal survival at 15°C, although lifespan was clearly different in each one of them (Table 1; Figure 2E). At 20°C, *A. obliqua* had the shortest lifespan (45.4 ± 6.5 days), unlike *A. serpentina,* which had the longest mean lifespan (82.2 ± 9.83 days) at this temperature (Figures 2G,H), although its mean maximal survival (170 ± 23.6 days) was reached at 15°C (Figure 2G; Table 1). At 30°C, *A. obliqua* and *A. ludens* exhibited similar lifespans (Figure 2J), but *A. obliqua* at this temperature had its longest lifespan (63.1 ± 4.51 days) (Figures 2I,J). Finally, at 40°C, the lifespan of all fly species was similar, although *A. striata* and *A. serpentina* lived more days than *A. ludens* (Figures 2K,L). The lifespan of all fly species was affected by extreme low and high temperatures (Figure 2; Table 1). *Anastrepha ludens* and *A. obliqua* resisted colder temperatures, and *A. serpentina* and *A. striata* hotter ones (Figure 2; Table 1).

**TABLE 1:** Female and male survivorship (mean ± SE, minimum and maximum values of days) of four *Anastrepha* species exposed to different temperatures in bioclimatic chambers. Results of the *t*-Test for Dependent Samples analysis to compare female and male survivorship of each species (t and *p-*values), mean ± SE of median survivorship of each species (females and males together) and Kruskal–Wallis test results (H and *p*-value) to compare mean of medians by temperature. *p*-values in bold denotes statistically clear differences.

	Species	Sex	5°C	10°C	15°C	20°C	30°C	40°C
**Mean ± SE**	*A. ludens*	F	18.2 ± 3.7	100.2 ± 8.8	212.6 ± 19.7	89.2 ± 11.8	43.1 ± 5.7	1.8 ± 0.4
		M	15.9 ± 3.6	86.9 ± 7.9	148.0 ± 10.0	95.7 ± 7.0	55.8 ± 6.1	1.8 ± 0.4
	t/*p-*values		2.28/0.083	1.32/0.255	3.22/**0.032**	-0.44/0.681	-3.75/**0.019**	0.11/0.913
	$\bar{X}$ Median	F + M	17.5 ± 4.9	105.9 ± 4.9	177.1 ± 22.7	101.4 ± 11.4	50.5 ± 5.98	1.8 ± 0.6
	*A. obliqua*	F	13.1 ± 2.0	32.2 ± 1.5	62.5 ± 14.6	43.9 ± 3.9	64.3 ± 7.5	2.2 ± 0.7
		M	10.9 ± 1.9	21.2 ± 5.1	41.2 ± 13.4	46.3 ± 2.9	62.4 ± 5.1	2.2 ± 0.7
	t/*p-*values		1.07/0.342	1.75/0.153	2.30/0.082	-0.72/0.509	0.16/0.873	-
	$\bar{X}$ Median	F + M	10.7 ± 1.59	14.1 ± 3.38	41.2 ± 19.66	45.4 ± 6.5	63.1 ± 4.51	2.2 ± 0.37
	*A. serpentina*	F	1.7 ± 0.3	13.6 ± 1.3	82.2 ± 13.3	66.9 ± 7.6	41.2 ± 4.0	3.2 ± 0.7
		M	1.8 ± 0.5	12.5 ± 0.6	49.0 ± 7.7	70.4 ± 5.6	37.4 ± 4.6	2.6 ± 0.4
	t/*p-*values		0.85/-0.201	1.16/0.307	1.95/0.122	-0.53/0.622	0.95/0.393	1.62/0.179
	$\bar{X}$ Median	F + M	1.2 ± 0.2	12.9 ± 0.99	52.6 ± 8.8	64.1 ± 10.6	39.5 ± 2.6	2.9 ± 0.55
	*A. striata*	F	3.6 ± 0.4	11.4 ± 1.0	62.3 ± 5.7	78.9 ± 5.9	36.8 ± 1.2	3.5 ± 0.3
		M	2.5 ± 0.2	16.4 ± 1.3	75.9 ± 6.4	80.3 ± 9.0	42.1 ± 1.4	3.1 ± 0.3
	t/*p-*values		2.04/0.110	-2.50/0.066	-1.86/0.136	-0.12/0.909	-6.77/**0.002**	0.79/0.469
	$\bar{X}$ Median	F + M	2.3 ± 0.3	11.3 ± 0.62	63.1 ± 13.3	82.2 ± 9.83	39.5 ± 2.2	2.7 ± 0.37
	**H/*p* value**		15.64/**0.001**	11.28/**0.007**	11.9/**0.007**	10.13/**0.017**	10.93/**0.012**	3.6/0.3074
**Minimum and maximum**	**Species**	**Sex**	**5 °C**	**10 °C**	**15 °C**	**20 °C**	**30 °C**	**40 °C**
	*A. ludens*	F	2–36	11–186	6–376	1–226	6–105	1–4
		M	1–37	7–183	7–344	1–196	2–132	1–6
	*A. obliqua*	F	3–30	1–96	1–162	1–135	4–126	1–3
		M	3–27	1–89	1–156	1–118	3–154	1–3
	*A. serpentina*	F	1–8	1–37	1–226	1–164	1–99	2–7
		M	1–7	1–35	5–176	3–159	1–97	2–6
	*A. striata*	F	1–9	1–45	1–176	6–181	1–71	1–9
		M	1–9	1–70	2–176	2–194	3–84	3–7

**FIGURE 3:**
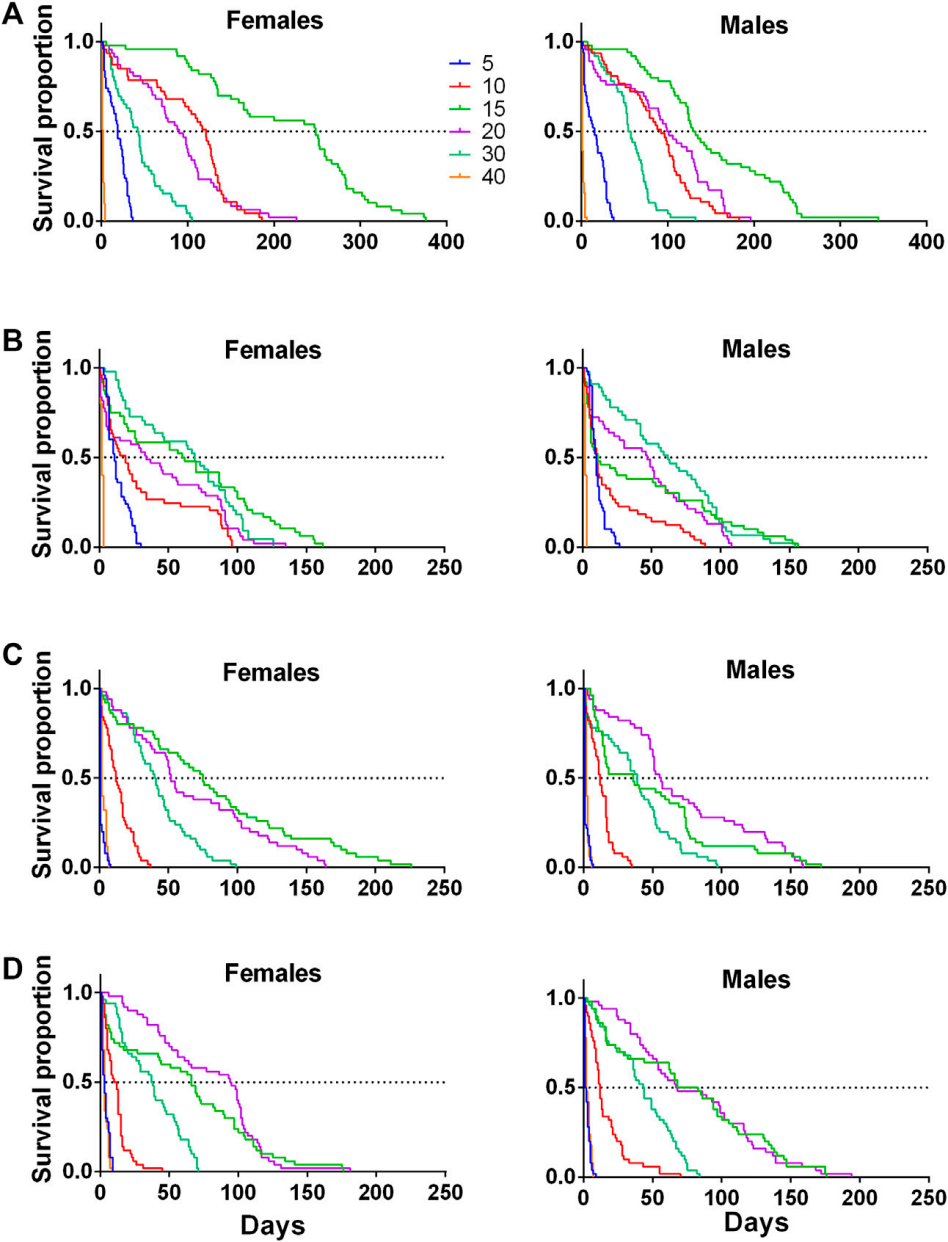
Kaplan-Meier survival curves of females and males of four pestiferous *Anastrepha* species exposed to different temperatures (5, 10, 15, 20, 30,40°C) in bioclimatic chambers **(A)** *A. ludens,* **(B)** *A. obliqua* **(C)** *A. serpentina* and, **(D)** *A. striata*).

Females lived more than males at 15°C in all species, except for *A. striata,* where females and males had similar lifespans (Table 1; Figures 3D). Approximately 50% of *A. ludens* females and males reached 100 days of age at 10 and 20°C (Figure 3A), while at 30°C the 50% of the lifespan of both sexes was almost half than at 15°C, i.e., 50 days (Figure 3A; Table 1). In *A. obliqua,* females and males lived longer at 30°C, but 50% of the population perished around day 62, practically a month less than *A. ludens* (Figure 3B; Table 1). In the case of *A. serpentina,* females exhibited a larger lifespan (around 80 days) than males at 15°C with a 50% lifespan when they reached 75 days, while males had the 50% of longest lifespan when they reached approximately 60 days (Figure 3C). Finally, *A. striata* females reached the highest 50% lifespan (approximately 100 days) at 20°C, while males only survived about 75 days at 15 and 20°C (Figure 3D; Table 1).

### Life expectancy/lifespan of female flies under laboratory conditions

**FIGURE 4:**
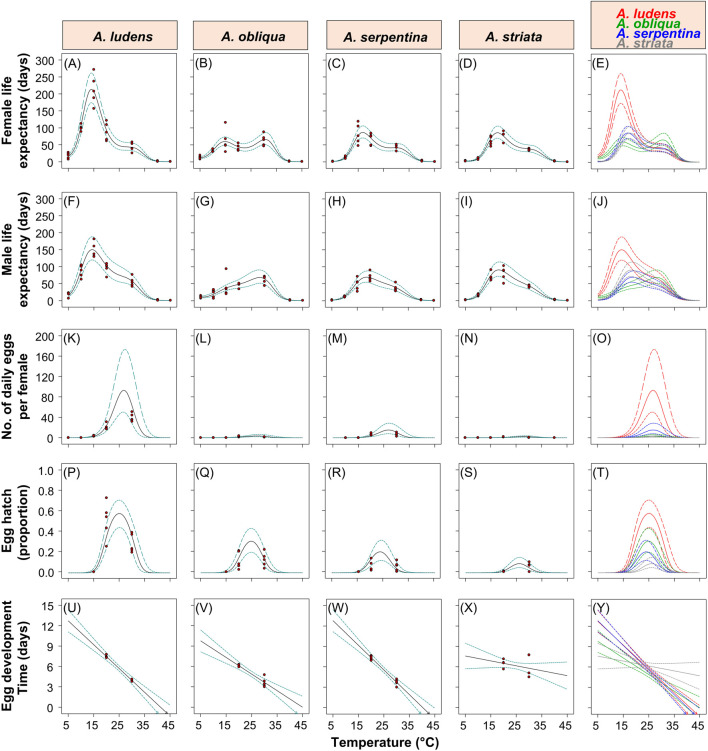
Polynomial models fitted to data on female **(A–E)** and male **(F–J)** life expectancy, the estimated daily egg production per female **(K–O)**, egg hatch **(P–T)** and egg development time **(U–Y)** of four *Anastrepha* species (from left to right *A. ludens*, *A. obliqua*, *A. serpentina* and *A. striata*) in response to an extreme increase in temperature. Solid lines indicate the models fitted to the data, and the dotted lines the 95% confidence intervals. Red points indicate the data points to which the models were fitted.

A sixth-order polynomial model indicated clear main and interaction effects of temperature and fly species on the average life expectancy of female flies (ANOVA full model: F = 195.69; df = 24, 115; *p* = 2.07^−76^; Supplementary Table S1). On average, females of *A. ludens* and *A. obliqua* lived longer periods (16.97 days in the case of *A. ludens* and 13.06 days in *A. obliqua*) than *A. serpentina* (1.68 days) and A. striata (3.71 days) at low temperatures (5°C) (Figures 4A–E). Females of *A. ludens* had the longest life expectancy of all four species, with an estimated maximum of ca. 214 days between 11 and 16°C (Figures 4A,E). *Anastrepha serpentina* and *A. striata* had similar estimated maximum life expectancies of ca. 87 days at temperatures between 14 and 20°C (Figures 4C–E), whereas the life expectancy of *A. obliqua* resembled a bimodal distribution in response to temperature with two estimated maximums of 60 and 66 days at temperatures between 12–17 and 27–33°C, respectively (Figures 4B,E). At 40°C, the species that lived the longest was *A. striata* with an estimated mean of 3.6 days of life expectancy, whereas at 45°C no fly species lived longer than an average of 1.1 days.

### Life expectancy/lifespan of male flies under laboratory conditions

A sixth-order polynomial model showed clear main and interaction effects of temperature and fly species on the average life expectancy of male flies (ANOVA full model: F = 156.21; df = 24, 115; *p* = 7.39^–82^; Supplementary Table S2). As was the case with female flies, males of *A. ludens* and *A. obliqua* lived longer periods (estimated mean of 14.56 days in *A. ludens* and 11.18 days in *A. obliqua*) than *A. serpentina* (1.67 days) and *A. striata* (2.63 days) at low temperatures (5°C) (Figures 4F–J). *Anastrepha ludens* males had the longest life expectancy of all four fly species with an estimated maximum of ca. 150.6 days between 11 and 18°C (Figures 4F,J). *Anastrepha serpentina* and *A. striata* had estimated maximum life expectancies of ca. 70–90 days at temperatures between 15 and 22°C (Figures 4H–J), whereas *A. obliqua* an estimated maximum of 80 days at temperatures between 26–31°C (Figures 4G,J). At 40°C, the species that lived the longest was *A. striata* with an average of 3.3 days of life expectancy, whereas at 45°C no species lived more than an average of 1.1 days (Figure 4J).

### Daily egg production per female under laboratory conditions

A cubic model indicated clear main and interaction effects of temperature and species on the daily mean egg production per female fly (ANOVA full model: F = 45.08; df = 12, 82; *p* = 5.4^–31^; Supplementary Table S3). No species laid eggs at temperatures between 5 and 10°C, and only *A. ludens* and *A. obliqua* laid eggs at 15°C with estimated means of 2.7 and 0.21 eggs per female per day, respectively (Figures 4K–O). *Anastrepha ludens* was the species that laid the largest number of eggs with an estimated maximum of 93 eggs per day per female at a temperature close to 25–27°C, whereas the maximums estimated for *A. obliqua*, *A. serpentina* and *A. striata* were ∼3.5, 15.3 and 2.4 eggs per day per female, respectively (Figures 4K–O).

**FIGURE 5:**
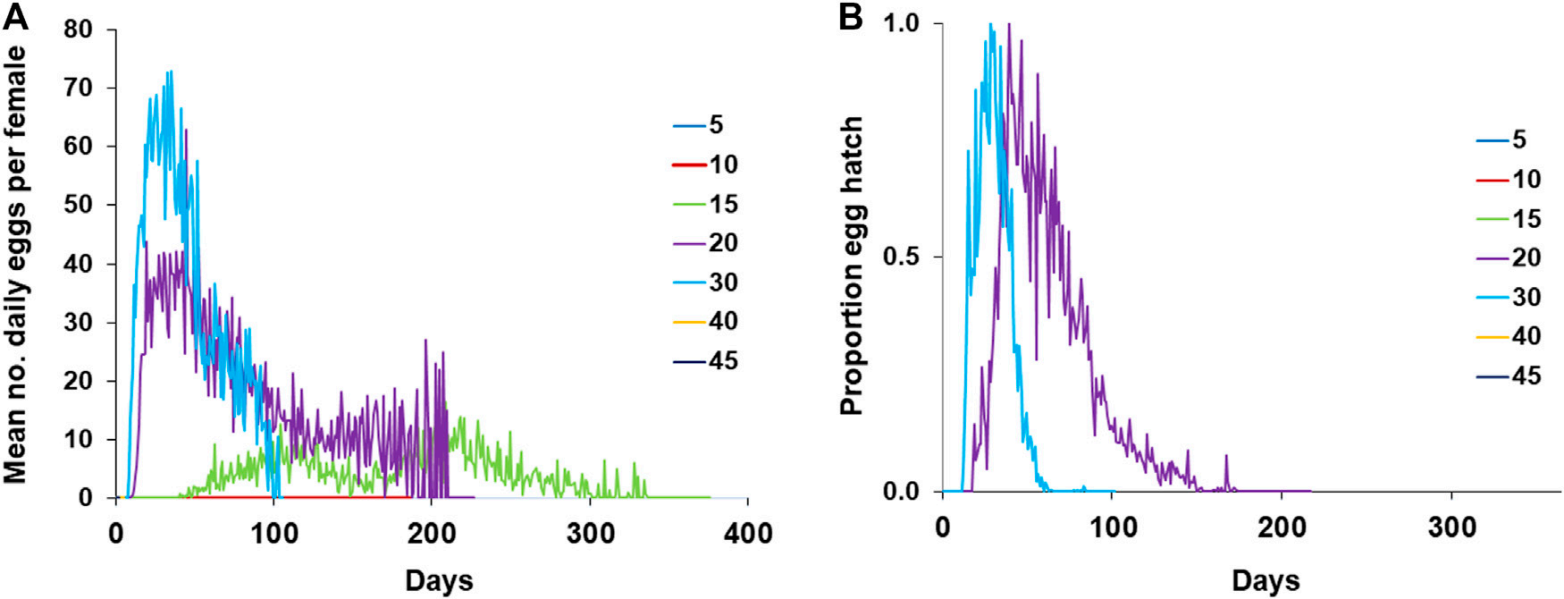
Reproduction parameters of *A. ludens* females exposed to different temperatures in climatic chambers **(A)** Fecundity; **(B)** Fertility.

Figure 5A presents the pattern of laid eggs by *A. ludens* females exposed to different temperatures showing that females only lay eggs at temperatures 15, 20, and 30°C. It stands out that the females exposed to 15°C regularly laid eggs from day 40 until day 335 (Figure 5A), although eggs did not hatch (Figure 5B). At 20°C, females laid eggs from day ten until day 209 with a peak between days 11 and 91 (Figure 5B). At this temperature, the percentage of eggs hatching fluctuated between 0 and 100 (Figure 5B). Finally, females exposed to a constant temperature of 30°C began to lay eggs at 8 days of age and finished at 102, 3 days before the last female of this treatment died.

### Egg hatch under laboratory conditions

A quadratic model indicated clear main and interaction effects of temperature and species on egg hatch (ANOVA full model: F = 17.06; df = 8, 41; *p* = 8.07^–11^; Supplementary Table S4). There was no hatching of *A. ludens*, *A. obliqua* and *A. serpentina* eggs at 15°C (in the case of *A. striata* there were no eggs to evaluate at this temperature). In the four fly species, the models fitted to data on egg hatch had an inverted “u” shape with the highest proportion of hatched eggs predicted by the models at temperatures between 21–29°C for *A. ludens* (proportion of 0.57) and *A. obliqua* (proportion of 0.30), 20–27°C for *A. serpentina* (proportion of 0.19), and 22–30°C for *A. striata* (proportion of 0.08) (Figures 4P–T).

### Egg development time under laboratory conditions

A two-factor interaction model (F = 33.94; df = 7, 29; *p* = 2.62^–12^) showed clear main and interaction effects of temperature and the fly species on egg development time (Supplementary Table S5). In *A. ludens*, *A. obliqua* and *A. serpentina*, egg development time decreased markedly as temperature increased from 20 to 30°C (Figures 4U–W), whereas in *A. striata* the same change in temperature had an unclear effect on egg development time (Figure 4X).

### Predicted life expectancy, egg production and egg hatch of *A. ludens* in hypothetical temperature conditions

Spring, summer, fall, and winter 2021 temperature estimates for Basel and Geneva are shown in Table 2 along with the hypothetical average temperature estimate for 2121 and predictions of life expectancy, egg production and egg hatch of *A. ludens* females for each predicted temperature. *Anastrepha ludens* females could live up to 170.62 days at an estimated summer temperature of 16.74°C in Basel, whereas at a spring temperature of 12.72 °C in Geneva the flies could reach a maximum lifespan of 198.32 days. Maximum egg production per female was estimated for summer temperatures of 16.74°C in Basel with an average of 5.23 eggs per female per day and 8.69 eggs per female per day at 18.38°C in Geneva (Table 2). Spring, fall, and winter temperatures were predicted to inhibit egg hatch in *A. ludens* (Table 2).

**TABLE 2:** The 2021 real temperature and predicted temperature for 2121 in two sites in Switzerland and the predicted life expectancy, egg production and egg hatch of *A. ludens* in such predicted temperatures.

Season	Site	Estimated temperature for 2021 (°C)	Predicted temperature for 2121 (°C)	Predicted life expectancy of female flies for 2121 temperature (Days)	Predicted production of eggs/female/day for 2121 temperature (Days)	Predicted egg hatch for 2121 temperature (proportion)
Spring	Basel	10.14	11.44	160.42	0.74	0.0
Spring	Geneva	11.42	12.72	198.32	1.25	0.0
Summer	Basel	15.44	16.74	170.63	5.23	0.07
Summer	Geneva	17.08	18.38	128.02	8.69	0.19
Fall	Basel	3.25	4.55	16.05	0.0	0.0
Fall	Geneva	4.44	5.74	20.30	0.01	0.0
Winter	Basel	0.14	1.44	ND	ND	ND
Winter	Geneva	1.71	3.01	ND	ND	ND

### Adult survival of four *Anastrepha* spp. under field conditions

**FIGURE 6:**
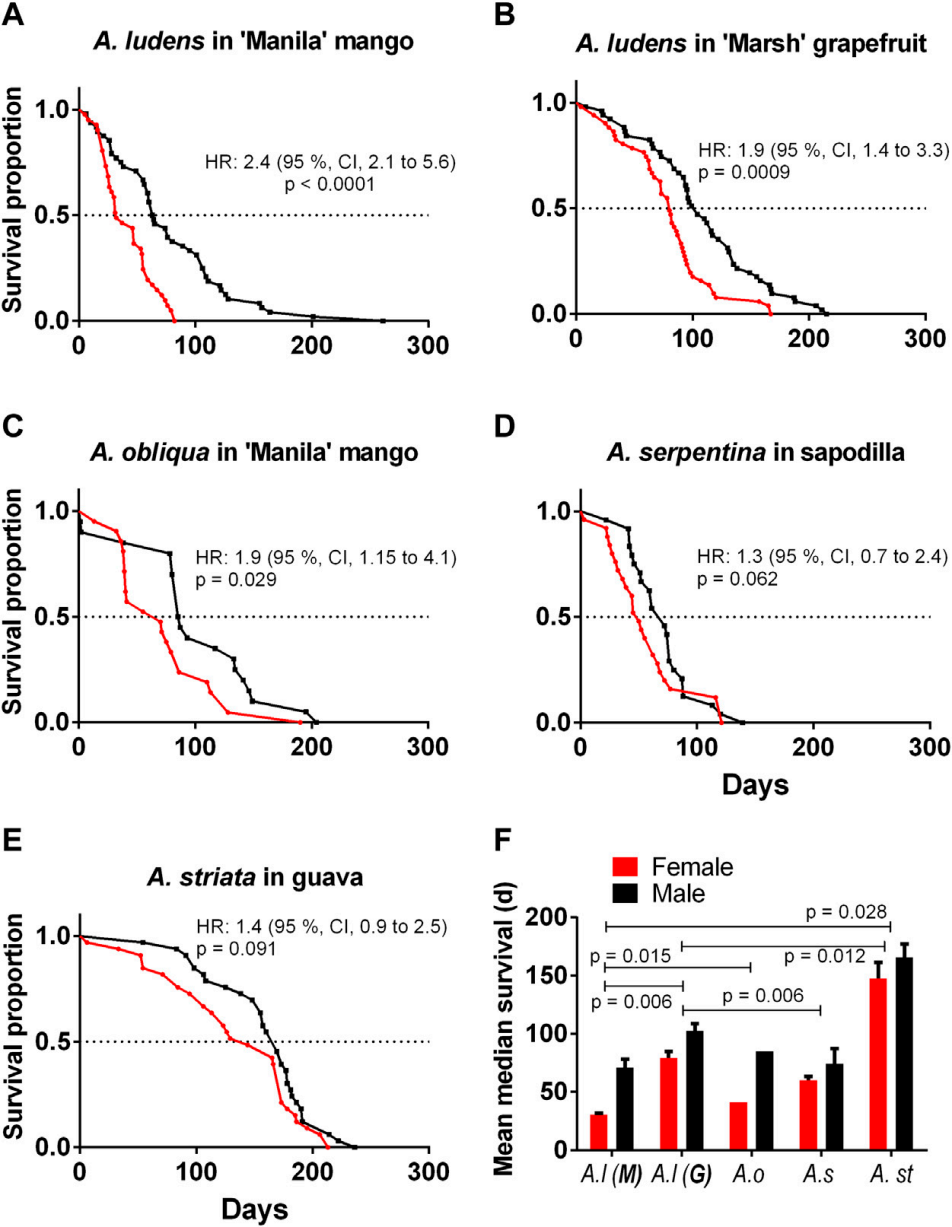
Kaplan-Meier survival curves of four pestiferous *Anastrepha* spp. under field conditions **(A)** *A. ludens* reared in mango in Apazapan, **(B)** *A. ludens* reared in grapefruit in Tolome; **(C)** *A. obliqua* reared in mango in Actopan; **(D)** *A. serpentina* reared in sapodilla in Apazapan; **(E)** *A. striata* reared in guava. The red lines show female survival, and the black ones male survival. **(F)** Mean ± SE of median survival (days) of females and males of the four *Anastrepha species*.

**FIGURE 7:**
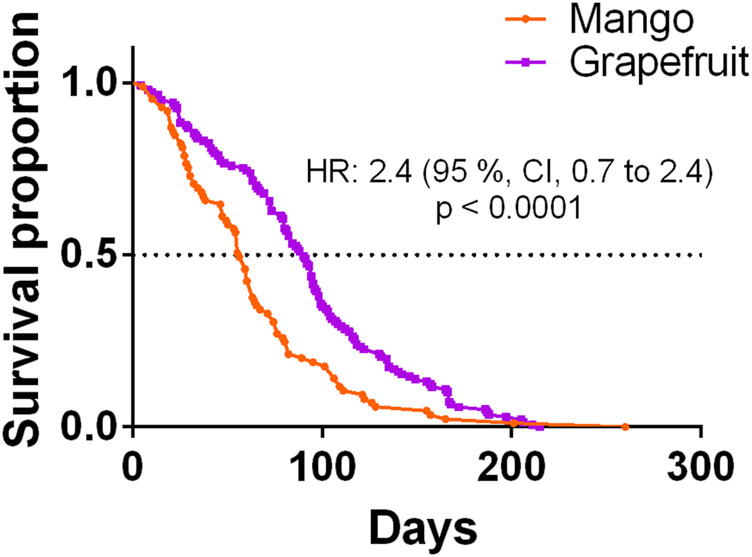
Kaplan-Meier survival curves of *Anastrepha ludens* females and males originating from two host plants (mango cv. ‘Manila’ and grapefruit cv. ‘Marsh’) under field conditions.

The mean lifespan of *A. ludens* in the field (Table 3) compared with the longest lifespan in the laboratory (15 °C treatment) (Table 1; Figures 2E,F) was ca. 70% lower for *A. ludens* stemming from mango and 46% lower than flies originating from grapefruit (Figures 6A,B). In the case of *A. obliqua,* the average lifespan in the field was 31% higher than the longest lifespan in the laboratory at 30°C (Table 1; Figures 2I,J and Figure 6C). Field and laboratory lifespans of *A. serpentina* were similar; laboratory flies at 20°C lived 5% more than field flies (Tables 1 and 3). Field *A. striata* lived 48% more than the laboratory flies at 20°C (Table 1 and Table 3; Figures 2G–H and Figure 6E). The lifespan of *A. ludens* females and males from ‘Marsh’ grapefruit in the field was higher than that observed in *A. ludens* from mango, and *A. serpentina* (Table 3). The lifespan of *A. ludens* developed in grapefruit was 87.7% higher than that observed in flies that developed in mango (Table 3, Figure 7). We found a statistically clear difference between the lifespan of males and females in all the studied species (Figure 6); all hazard ratios comparing survival curves show that females lived less than males in the field.

**TABLE 3:** Means of median, maximum, and minimum survival days ±SEM of the different *Anastrepha* spp. in the field (females and males together).

Survival parameters in days	*A. ludens* (mango)	*A. ludens* (grapefruit)	*A. obliqua* (mango)	*A. serpentina* (sapodilla)	*A. striata* (guava)	F _4, 30_ (*p*-value)
Median (±SE)	53.6 ± 3.7	95.2 ± 4.7	92.3 ± 5	60.8 ± 8.3	159 ± 8.27	23.58 (0.0001)
Maximum (±SE)	173.8 ± 38	190 ± 10.6	167.4 ± 22.8	127 ± 6	211.5 ± 11.1	
Minimum (±SE)	8.6 ± 3.1	18.1 ± 5	14.4 ± 8	30.3 ± 6	49 ± 16	

### Development time of *Anastrepha* spp. immature stages under natural field conditions

**FIGURE 8:**
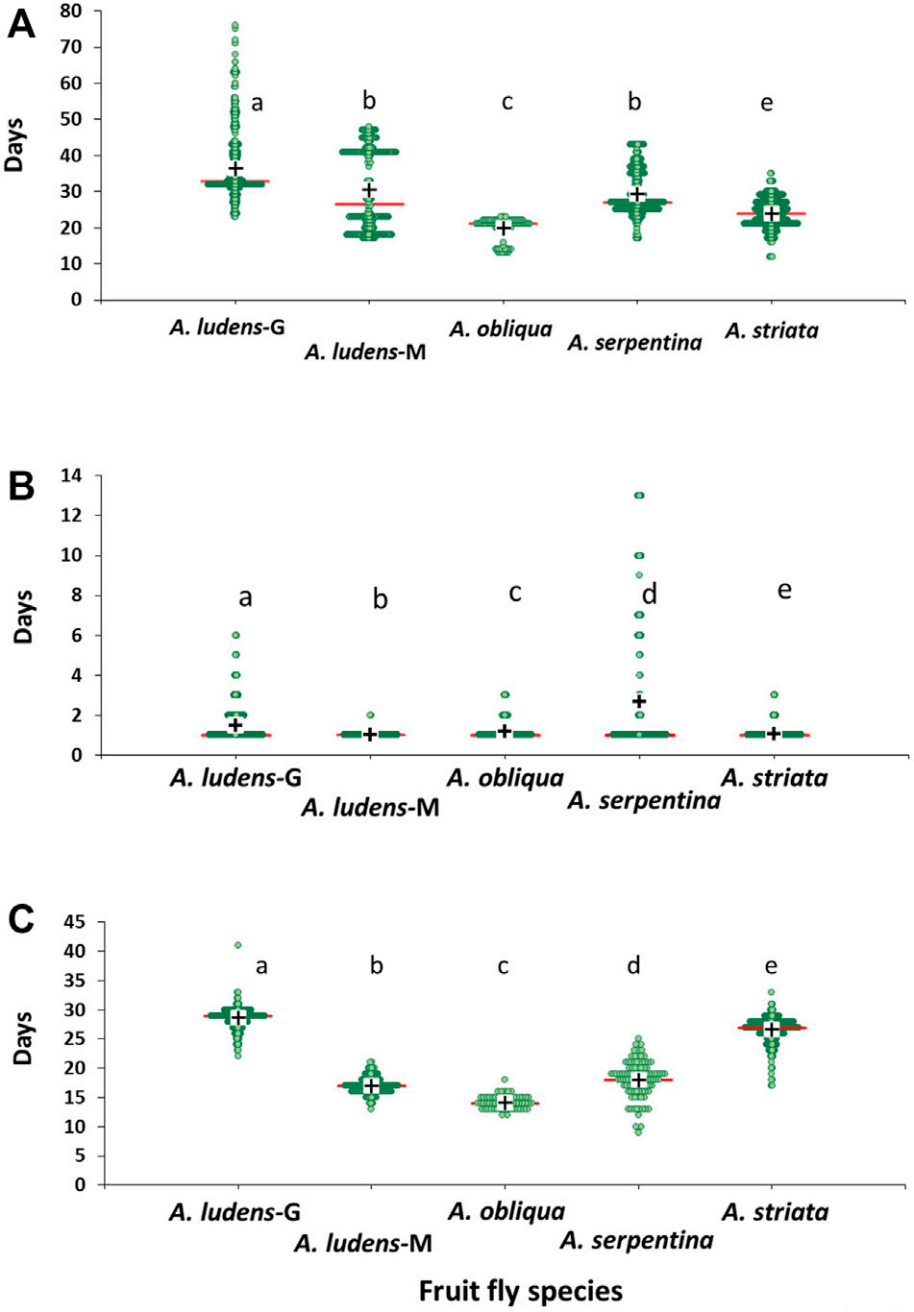
Development time in days of immature stages of four *Anastrepha* species reared under field conditions **(A)** Egg-third instar larvae; **(B)** Third instar larvae-pupae; **(C)** Pupation period. Green points show dispersion data, red horizontal lines show the median and black crosses the mean of days. *A. ludens*-G (from grapefruit), *A. ludens*-M (from mango).

We found statistically clear differences in the development time from egg to third instar larva among the studied *Anastrepha* species (H = 922.38; df = 4, 2,101; *p* < 0.0001; Figure 8A). *Anastrepha obliqua* had the shortest development time with an estimated mean (±SE) of 19.8 ± 0.34 days, whereas the longest development time was observed in *A. ludens* from grapefruit with an estimated mean of 36.4 ± 0.32 days, followed by *A. ludens* from mango with 30.5 ± 0.64 days (Table 4; Figure 8).

**TABLE 4:** Mean (±SE), minimum and maximum values for development times (in days) of immature stages of four *Anastrepha* species reared under field conditions *A. ludens*-G (from grapefruit) and *A. ludens*-M (from mango).

Fruit fly species	Eggs-third instar larvae			Larve-pupae			Pupation		
	Mean	Min	Max	Mean	Min	Max	Mean	Min	Max
***A. ludens*-G**	36.41 ± 0.32	23.00	76.00	1.48 ± 0.03	1	6.00	28.64 ± 0.06	22.00	41.00
***A. ludens-*M**	30.54 ± 0.64	17.00	48.00	1.01 ± 0.00	1	2.00	16.96 ± 0.10	13.00	21.00
***A. obliqua***	19.81 ± 0.34	13.00	23.00	1.19 ± 0.05	1	3.00	14.18 ± 0.14	12.00	18.00
***A. serpentina***	29.40 ± 0.33	17.00	43.00	2.66 ± 0.20	1	13.00	18.06 ± 0.31	9.00	25.00
***A. striata***	23.89 ± 0.15	12.00	35.00	1.07 ± 0.01	1	3.00	26.70 ± 0.09	17.00	33.00

We found statistically clear differences in the time that the larvae of the different *Anastrepha* species took to pupate (H = 210.02; df = 4, 1971, *p* < 0.0001). *Anastrepha serpentina* larvae took an average (±SE) of 2.7 ± 0.20 days to pupate with a maximum of 13 days, followed by *A. ludens* reared on grapefruit (1.5 ± 0.03 days) (Table 4; Figure 8B).

Finally, we found statistically clear differences in pupation time among the four *Anastrepha* species studied (H = 1,032.77; df = 4, 1,552; *p* < 0.0001). *Anastrepha ludens* reared in grapefruit had the longest pupation time (28.64 ± 0.06) whereas the shortest time was observed in *A. obliqua* (14.18 ± 0.14) (Table 4; Figure 8C).

### Temperature and humidity patterns in the field

**FIGURE 9:**
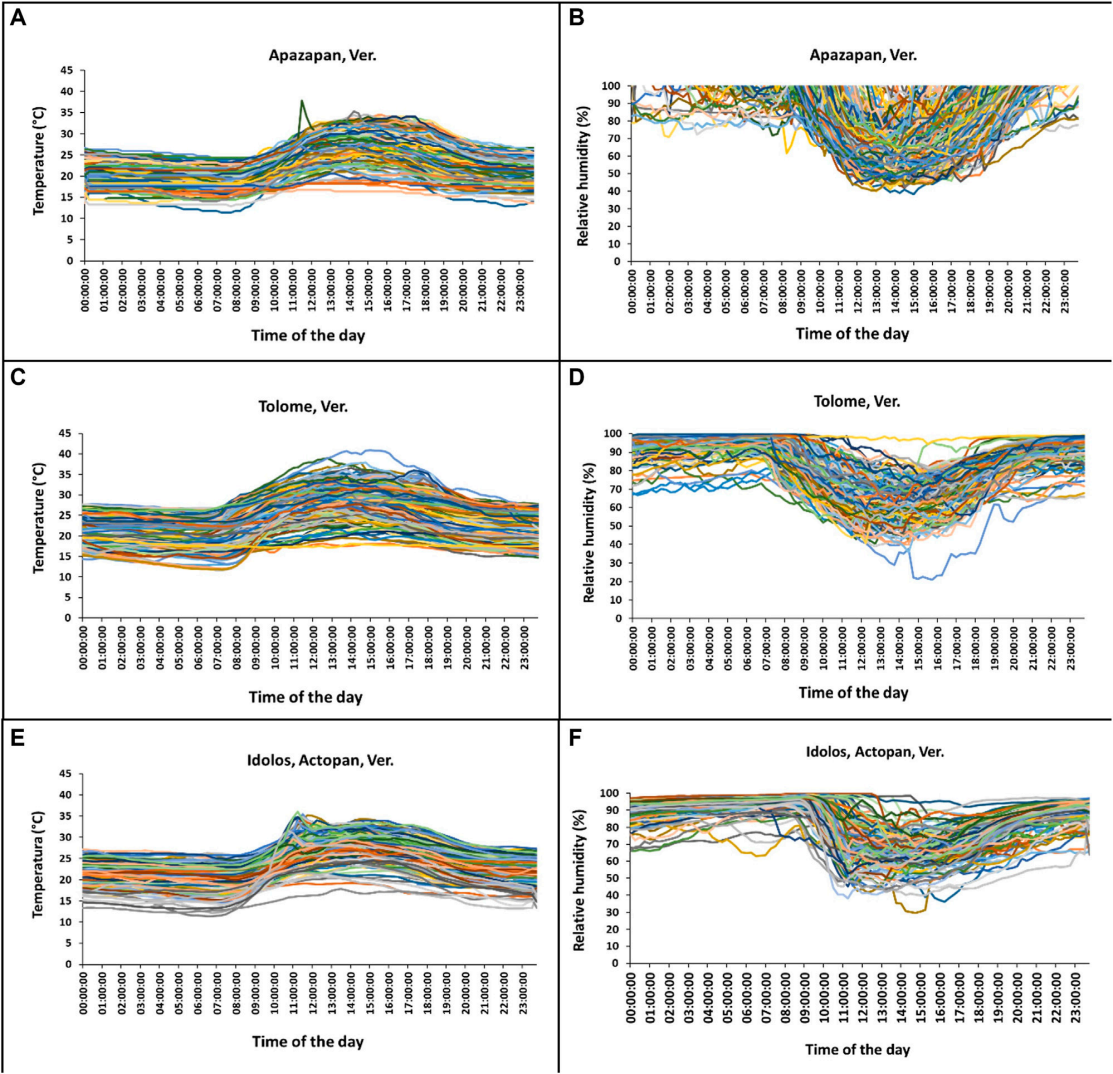
Mean temperature and RH per hour of each day throughout the experiment in **(A–B)** Apazapan (mango, sapodilla, and guava area) **(C–D)** Tolome (citrus area), and **(E–F)** Idolos, Actopan (mango area) in Veracruz, Mexico.

There was a clear difference in temperature and relative humidity (RH) across time among hours in any given day and over days (Figure 9; Table 5). Temperature and relative humidity patterns were contrasting: when the highest temperatures were measured, relative humidity values dropped to their lowest points. In all sites, the temperature and RH patterns were similar, with variations along the day and the season (Figure 9). The lower temperatures were recorded between 00:00–07:00 h, and between 20:00 and 23:00 h at Tolome and Actopan (Figures 9C,E). In the case of Apazapan, the lower temperature occurred from 00:00 to 09:00 h and between 20:00 and 23:00 h (Figure 9A). In Tolome, the place where *A. ludens* naturally infests grapefruit, recorded temperatures reached more than 40°C at 15:00 h in April, but in February, the temperature was 11°C at 07:00 h (Figure 9C). In Actopan, the site where *A. obliqua* and *A. ludens* infest mangoes, the maximum temperature reached 36°C in October, whereas the minimum was 12°C in January (Figure 9E). At this site, the temperature pattern presented two peaks, one in October and the other in September (Figure 9E). In Apazapan, a mango and sapodilla producing area, the temperatures fluctuated between 11°C (November) and 38°C (September) (Figure 9A). In Tolome, the site with the highest temperatures recorded, the RH dropped to 21% in April, which is the dry season (Figure 9D). In general, the lowest humidity percentages fluctuated between 40 and 80% and occurred between 10:00 and 17:00 h, which coincides with the hours of the day when temperatures were at their highest peaks. In Apazapan and Actopan, the highest percentages of RH (80–100%) were recorded between 00:00 and 09:00 h and between 20:00 and 23:00 h for the case of Actopan (Figures 9B,F). In the case of HR, Apazapan presented a very variable pattern between 09:00 and 20:00 h.

**TABLE 5:** Climatic data of field study sites in Veracruz, Mexico.

Climatic data	Apazapan (mango, guava and sapodilla area)			Tolome (citrus area)			Actopan (mango area)		
	Mean	Min	Max	Mean	Min	Max	Mean	Min	Max
**Temperature (°C)**	23.5	11.8	34.4	25.16	11.6	40.92	23.9	11.3	36.0
**RH (%)**	92.4	38.4	100	82.8	21.4	100	83.1	29.5	100
**Altitude (masl)**	408	40	96						

## Discussion

In this study, we addressed a key topic of research in insect developmental biology and physiology that is critical for decision-making in the management of pestiferous insect invasions and the understanding of insect ecology in the face of global warming (Wagner et al., 1984; Samayoa et al., 2018; Lisbôa et al., 2020; Schneider et al., 2021). Our results show clear patterns indicating that some of the four species studied will suffer more under a global warming scenario and that as predicted, *A. ludens* lived significantly longer under controlled laboratory conditions when compared to highly variable field conditions exerting metabolic challenges (Table 1 and Table 3; Figure 9). Interestingly, the two species belonging to the more basal *serpentina* species group (*A*. *striata* and *A*. *serpentina*, Norrbom, 2002) survived more days as adults under the constant and extreme temperature of 40°C (Figures 2K,L), and in the case of *A. striata*, it was the species in which adults lived more time under variable conditions in the field (Table 3), implying that these species will better cope with rising temperatures granted their host plants also adapt to these temperatures. Individuals of these two species also suffered the most when exposed to the low temperatures of 5 and 10°C (Figures 2A–D). Contrary patterns were observed in the two representatives of the *fraterculus* species group (*A*. *ludens* and *A*. *obliqua*). At a constant temperature of 45°C, adults, independent of species, only managed to survive a single day, likely due to thermal shock. We note however that in nature adult flies can move to cooler places during the hottest hours of the day (Aluja and Birke, 1993) and that the air temperature is not the same as the temperature in the pulp and thus could likely survive for longer periods as adults. In the case of *A*. *ludens*, the species exhibiting the widest distribution margin in nature when considering altitude above sea level, our data clearly show that its adult survival rate peaked at 15°C (Figures 4A,F), which means that under a global warming scenario, it is likely that the altitudinal distribution range of this species will shift from lowland tropical regions to higher altitude ones, using new hosts along the way. At the same low temperature, females laid large numbers of eggs, but none hatched. In contrast to *A*. *striata* and *A*. *serpentina*, *A*. *ludens* survived for up to 40 and 185 days, respectively, at 5 and 10 °C degrees. Surprisingly, *A*. *obliqua*, a species that is found in warm, tropical environments, fared much better than *A*. *striata* and *A*. *serpentina* at 5 and 10°C degrees, respectively (e.g., females survived up to 30 and 125 days, respectively). In all species, egg hatch was first recorded at 20°C but it is possible that the eggs that did not hatch at 15°C were viable and would have hatched if temperature conditions changed (i.e., if eggs were moved from an environmental chamber under 15°C to one under 20 or 30°C). Importantly, field data, under highly variable temperature and relative humidity conditions (Figure 9), yielded similar overall patterns among the four species studied (compared to the laboratory studies), but with significantly shorter survival rates in the cases of *A. ludens* and *A. serpentina* (Table 1 and Table 3). The latter, likely due to an increased metabolic wear/exhaustion caused by the need to cope with the highly variable environmental conditions (e.g., Moloń et al., 2020). Also, field conditions, flushed out an interesting difference in survival between sexes in *A*. *ludens*. In what follows, we expand on the results of our modelling approach and their implications related to global warming, and discuss our findings considering the physiological/metabolic processes possibly regulating adaptation to varying temperatures.

Our modelling approach allowed us to determine how the life expectancy, fecundity, fertility, and egg development time in four *Anastrepha* species, some of which infest the fruit of various plant species including commercial mango and citrus, differentially vary as a function of a wide range of temperatures from 5 to 45°C (Figure 4). The same approach allowed us to make predictions on the life expectancy, fecundity, and fertility of *A. ludens* females in hypothetical invasion scenarios to temperate areas as a consequence of global warming. We used Switzerland as an illustration of a European country which already has been invaded by an American tephritid fruit fly (Walnut Husk Fly, *R. completa*, Aluja et al., 2011), and where commercial apple (*Malus × domestica* Borkh.) production could be severely affected by an *A. ludens* invasion (Birke et al., 2013; Aluja et al., 2014). Based on our models and analyses, we calculated that in the case of a future increase in temperature and expansion of *A. ludens* to temperate areas, females could potentially live more than six months in spring and summer temperatures of 11.44–18.38°C (Figure 4A, Table 2). Over three months at such temperatures, a single *A. ludens* female could potentially oviposit up to 792 eggs and leave an estimated larval offspring of 150 individuals (i.e., 8.69 eggs per day and a hatching rate of 19%, Table 2). Although our study did not directly evaluate the effects of temperature on larval development time, it is well known that in *A. ludens* (Messenger and Flitters, 1957; Thomas, 1997; Aluja et al., 2010) and other tephritid pest species (Samayoa et al., 2018; Huang et al., 2020) temperature is a critical factor that affects development. In this study, we observed that *A. ludens* larvae could live almost three months inside grapefruit (Figure 8A) in a field site where the overall mean temperature was ca. 25.16°C, but that during winter can drop to 10°C. Cold winter temperatures are known to extend pupal development time of *A. ludens* (Thomas, 1997), and the minimal lethal time (LT) for 99.9968% mortality of phanerocephalic pupae and pharate adults at a constant temperature of 1.1°C was estimated to be 20.8 and 20.2 days, respectively (Hallman, 1999). This means that *A. ludens* individuals likely activate physiological mechanisms that allow them to extend developmental periods in response to cold temperatures (a topic discussed later). In fact, our study showed that the egg development time of *A. ludens*, *A. obliqua* and *A. serpentina*, takes longer at low temperatures than at high temperatures (Figures 4U–W). This could help flies to withstand cold winters in the case they invade temperate areas. But given that temperature and RH conditions in nature fluctuate significantly (Figure 9) caution should be applied when reaching conclusions from experimental data using constant temperatures. However, our study provides a valuable way to address the issue of thermal thresholds in insects using predictive models generated from experimental data under controlled laboratory conditions, which together with climatic predictions and life history theory of the studied species, contributes to improving our understanding of insect developmental physiology. But as underlined by Hagstrum and Milliken (1991) and Fischer et al. (2011), future studies in the laboratory should consider varying temperatures to predict life histories of insects more accurately in nature.

Our results confirm old findings (Messenger and Flitters, 1957) on the negative effect of high temperatures on the survival of adult flies (Figures 2–4). An extreme increase in temperature in the current range of distribution of flies could force them to move to temperate zones. In fact, recently, we have been documenting infestations of *A. ludens* in commercial apple orchards growing in temperate areas of Mexico where they were not found before (MA, pers. obs.). In addition, extremely hot temperatures were recorded in Mexico last year (2021) in Hermosillo, Sonora, reaching more than 45 °C in some places (NASA, 2022). Hermosillo is currently a fruit fly pest-free zone (Gutiérrez-Ruelas et al., 2013), therefore our data will help managers gauge strategic decisions in this area of the country as we now have reliable information on the upper thermal limits of *A*. *ludens*. The same applies to other areas of the world such as for example Northern Africa, many parts of Asia and Australia where temperatures easily reach 45°C. We note however, and as documented by Aluja and Birke (1993) in the case of *A*. *obliqua*, adult flies move back and forth during the day between habitat units, in this case, a tropical plum tree (*Spondias purpurea* L.) devoid of leaves at the time of study, and a densely foliated mango tree, using the latter as a refuge to deal with the extremely hot temperatures at midday (close to 45°C). So adult flies will likely live for much longer periods as observed here in the laboratory at 40 and 45°C as there they had no place to escape. In addition, it must be considered that as is the case with extremely low temperatures (discussed before), the temperature inside fruit is lower than the air temperature where the tree grows. So, the survival of larvae will be higher when protected by the fruit, compared to adults exposed to very high air temperatures granted they do not find refuge in a densely foliated tree or crevice in for example rocky walls or dry riverbeds. But the latter depends on the size to the fruit and the degree of leaf cover in the tree because Aluja and Birke (1993) also documented the fact that *S*. *purpurea* fruit, which have a very large seed and as a result very little pulp and a thin skin, can heat up considerably forcing larvae to jump out of the fruit dropping up to 10 m to the ground to bury themselves and pupate (if an ant does not kill them before (Aluja et al., 2005)). In the case of immature stages, surviving at high temperatures can be more complex. Pucci et al. (1981) concluded that most of the eggs and larvae of *B. oleae* die when the olive fruits reach maximum temperatures of 36°C for 1 week. The air temperature, the size of the fruit and its location on the tree or the ground influence the internal temperature of the fruit and the survival of immature stages (Sivinski et al., 2007). Eggs cannot avoid high and mortal temperatures inside some fruit, but females can diminish the risk of desiccation by avoiding laying eggs in sun-exposed sites on the fruit (Guillén et al., 2022) and larvae can move to fresher areas within the fruit (Sivinski et al., 2007), or as noted before crawl out of the fruit and jump to the ground.

One of the strengths of our experimental approach is that we studied all four species under controlled conditions using a fixed temperature regime that spanned over a wide range of temperatures, as well as under highly variable natural conditions. This approach enabled us to flush out interesting differences between males and females in most species (Figure 6) that point to physiological mechanisms that differ between the sexes (details follow). It also allowed us to determine that, for example, *A*. *ludens* individuals, can survive as adults up to 200 days in the field and up to 370 days in the laboratory under a constant temperature regime of 15°C. Notably, females of this species can lay viable eggs after 150 days of age (Figure 5B) at 20°C and lay many eggs at 15°C but that do not eclose (Figures 5A,B). Low and high temperatures are known to decrease sperm production and viability in *Drosophila*, which can lead to sterility in males (David et al., 2005). Perhaps *Anastrepha* flies maintained at low temperatures in our study suffered from damage to the male reproductive system, which in turn could have reduced mating frequency or sperm quality (Meats and Fay, 2000; Walsh et al., 2019). In contrast, when flies were subjected to high temperatures, the production of eggs in the ovaries was accelerated as was the case with the olive fly, *B*. *oleae* (Wang et al., 2009), but likely at the cost of high energy expenditure (Novoseltsev and Novoseltseva, 2013) and a reduction in lifespan. All this has important implications for the management of fruit flies applying the Sterile Insect Technique as adults are many times chilled prior to release in the field (Hernández et al., 2010). Another relevant effect of temperature on the biological traits of flies is related to the size of the eggs. Three different scenarios have been observed in insects: 1) at constant temperatures, the size of the eggs remains without significant changes; 2) at low temperatures, females generally produce fewer numbers of eggs, but the size of the eggs is larger (Fox and Czesak, 2000; Fischer et al., 2011; Xu et al., 2012); 3) at high temperatures females produce a greater number of eggs but with a relatively small size (Fox and Czesak, 2000; Fischer et al., 2011). Under field conditions, egg size is expected to be highly variable because of the influence of fluctuating temperatures and food availability (Xu et al., 2012). Finally, we would like to highlight the differences observed in the field in the case of *A*. *ludens* infesting two different hosts (grapefruit and mango). Given that these fruits do not overlap because of their different fruiting phenologies, we were not able to conduct the study simultaneously and thus attribute a definitive host effect under equal environmental conditions. But the patterns observed were very interesting. Both sites had overall similar temperatures and RH (Figure 9; Table 5) and, in both sites, insects were exposed to low (ca. 11°C) and high temperatures (ca. 38°C). However, the lifespan of adults and development time of immatures were significantly longer in grapefruit than mango (Figure 8). The difference, independent of host type, was the moment at which immature stages experienced the cold stress. In grapefruit, immature stages experienced cold temperatures from December to February. Notably, the development time from egg to larvae extended for some individuals over almost 80 days. Under more favorable environmental conditions, this period covers ca. 12 days (five for egg incubation and seven for larvae development; Aluja, 1994). In contrast to what occurred in grapefruit, in mango, insects were exposed to cold temperatures at the end of their adult life (not as eggs or larvae). In other Diptera with facultative diapause, it has been reported that early-instar larvae, depending on the environmental cues received, prolong the duration of larval or pupal stages (Denlinger, 2002). In *A. ludens* a facultative diapause has not been reported, but our observations here (i.e., extremely long egg to larvae periods), suggest that it could be a mechanism triggered to deal with adverse (i.e., cold) temperatures. We note too that subtle changes in temperature or relative humidity can have subtle effects on immature development time as recently documented by Manenti et al. (2021) and references therein working with five *Drosophila* species with different thermal niches. These authors were able to document the fact that several species of the same genus “can show substantial differences when developing at fluctuating temperatures not always predictable by development at comparable constant temperature (25°C)”. These results relate nicely to ours as we also observed different development patterns in for example *A*. *ludens* developing in different fruit in two times of the year, highlighting the fact that studies under variable environmental conditions are crucial to fully understand the abiotic factors driving immature development. For further discussion on this critical issue, please see Parmesan (2006), Jaworski and Hilszczański (2013) and Ketola and Saarinen (2015).

Among the most critical physiological responses to temperature changes in insects, the following stand out: 1) sensory responses, 2) changes in metabolic rates, 3) responses to heat stress mediated by HSP proteins, and 4) modulation of hormones that culminate in modifications in development time and behavior (González-Tokman et al., 2020). In addition, phenotypic attributes such as foraging, regurgitation and mating behavior, abdominal ventilation, cuticular biopolymers, and body size help insects to deal with extreme temperatures (Perez and Aron, 2020). Some plastic physiological responses can counteract the mechanical, structural, and functional challenges in cells, mainly in cell structure, protein activity, and energetic balance. Changes in cell membrane composition have been described to avoid fluidity modifications and interchange of proteins that have higher flexibility in response to extreme temperatures (Koštál et al., 2007). Besides, dehydration has been described as a response to extreme temperatures. The water loss in the insect body can decrease the risk of ice crystallization and damage to cell structure (Toxopeus and Sinclair, 2018). In the case of flies depending on the species, the crystallization temperature is between 0 and -65°C (Lee, 1991). The synthesis of bioamines and other small molecules with cryoprotectant qualities as well as glycerol and trehalose can counteract the protein stress response to heat shock. In this respect, there is evidence of significant effects in transcriptome and metabolome organization from *Drosophila melanogaster* Meigen during cold acclimation (MacMillan et al., 2016). Under laboratory conditions, the effects observed on primary functions may be due to the consequence of phenotypic qualities and the physiological and metabolic plasticity that each species have. In *Drosophila* an increase in temperature causes the energy stored in the form of fat in adipose tissue cells to be metabolized much faster and once the energy reserves are depleted, cells get damaged by apoptosis (i.e., programmed cell death) (Klepsatel et al., 2016). In our study, *A*. *ludens* and *A*. *obliqua* were the species that lived shorter periods at high temperatures, which may reflect a depletion of their adipose tissue reserves and cellular damage. Our results point to a division between the *fraterculus* (*A. ludens* and *A. obliqua*) and *serpentina* (*A. serpentina* and *A. striata*) groups in their resistance to survive extreme temperatures (Figure 2). Thus, identifying the biochemical and physiological mechanisms behind such responses promises to be a fruitful endeavor.

We found that *A. ludens* and *A. obliqua* adults kept under experimental laboratory conditions, tolerated low temperatures of 5 and 10°C for longer periods than *A. striata* and *A. serpentina* (Figure 2; Table 1). Causes of cold injury include dehydration, osmolyte concentration, disturbance in homeostasis, oxidative stress, energy loss, protein dissociation and/or denaturation, and cell damage (Privalov, 1990; MacMillan and Sinclair, 2011; MacMillan et al., 2015a; MacMillan et al., 2015b; Koštál et al., 2016). There are several molecular mechanisms that insects exhibit in response to cold stress, including synthesis of cryoprotective molecules such as polyhydric alcohols (glycerol, sorbitol, mannitol, erythritol, and myo-inositol), sugars (glucose and trehalose) (Koštál et al., 2007; Doucet et al., 2009; Toxopeus et al., 2019) and aminoacids (arginine, asparagine, glutamine, and proline) (Michaud and Denlinger, 2007; MacMillan et al., 2016; Olsson et al., 2016). Other mechanisms include changes in the profile of membrane phospholipids (Enriquez and Colinet, 2019; Trenti et al., 2022), induction of antioxidant enzyme activities (Joanisse and Storey, 1996), and the gene expression induced by cold such as heat shock proteins (HSP: HSP22, HSP23, HSP26, HSP67, and HSP70Bbb, *etc.*), circadian rhythm and metabolism related genes (*Frost*, *smp-30, Starvin* and *hsr-omega*) (Sejerkilde et al., 2003; Sinclair et al., 2007; Colinet et al., 2010; Vesala et al., 2012; King and MacRae, 2015; MacMillan et al., 2016). The function of HSPs is dependent on the physiology of a particular insect and environmental conditions. In response to stress, small HSPs bind to denaturing proteins and prevent the irreversible protein aggregation, while ATP-dependent HSPs are focused on the protein refolding and/or degrading and restoration of homeostasis (Basha et al., 2012; Clare and Saibil, 2013; King and MacRae, 2015). Directed studies are needed to determine if the species of the *fraterculus* group studied here activate some of these mechanisms to tolerate cold temperatures for longer periods, and if the representatives of the *serpentina* group lack them.

The constant temperature of 15°C prevented egg hatch (Figure 5B). In places where seasonality is pronounced, *D. melanogaster* females inhibit egg production in cold temperatures activating an “ovarian diapause", characterized by the reduction of vitellogenin in the eggs and the absence of ovarian development (Denlinger, 2002; Williams and Sokolowski, 2009). We note that the absence of egg hatch observed at 15°C in *A*. *ludens*, does not necessarily imply that females were infertile as it is known that a short exposure to optimal temperatures after a cold shock can allow the recovery of insects from possible injuries caused by extreme cold (Colinet et al., 2015).

On the other hand, *A. striata* and *A. serpentina* were more tolerant to high temperatures than *A. ludens* and *A. obliqua* (Figure 2K; Table 1). Several studies have shown that tropical ectotherms, which are faced with almost permanent high temperatures, have a lower tolerance for heat than temperate ectotherms and tend to seek shelter or move to places that protect them from heat stress (Kearney et al., 2009; Sunday et al., 2014). In Mexico, *A. ludens* and *A. obliqua* are found from 0–2000 masl and 0–1,400 masl, respectively, a much wider altitudinal distribution range when compared to *A. striata* and *A. serpentina*, which occupy ranges between 0–1,200 masl (M.A, unpublished data). In a comparative study between *B. correcta* and *B. dorsalis*, two species with partially overlapping distributions in China, it was shown that *B. correcta* exhibited higher survival rates at 39–41°C, or after short exposures to 45°C than *A*. *dorsalis*. Consistent with this, HSP70 and HSP90 transcripts were identified in *B. correcta*, but not in *B. dorsalis* (Hu et al., 2014)*.* HSP70 was also overexpressed after heat stress in *R. mendax* (Teixeira and Polavarapu, 2005), and both, HSP70 and HSP90, were involved in the heat response of *R. pomonella* (López-Martínez and Denlinger, 2008). Interestingly, differences between the sexes have been found. For example, Bauerfeind et al. (2018) found higher levels of HSP proteins in females than in males of the yellow dung flies (*Scathophaga stercoraria* (L); Diptera: Scathophagidae), but males performed better than females in response to extreme temperatures. Our results show that the mean lifespan of males in the field was significantly longer than the one of females in the case of species of the *fraterculus* group (Figure 6). In the case of our laboratory studies, we found differences between females and males of *A. ludens* at 15°C and, in *A. ludens* and *A. striata* at 30°C (Table 1). At 15°C, *A. ludens* females lived more than males, and at 30°C in both *A. ludens* and *A. striata*, males lived more than females. Perhaps *A*. *ludens* females triggered a faster response to thermic stress *via* a more robust expression of HSP´s compared to males. This could explain the longer survival of *A. ludens* females at a constant temperature of 15°C. Under highly variable field conditions, *A*. *ludens* females lived for significantly shorter periods than males (independent of host origin) possibly due to a higher energetic and physiological cost in activating response mechanisms to thermal stress. Energy costs associated with tolerance to heat have been studied in *D. melanogaster* larvae. For example, transgenic flies with overexpression of HSP70 are more tolerant to higher temperatures but with the cost of reduced growth, survival, and egg hatch when compared to wild-type flies (Krebs and Feder, 1997). More recently, Moloń et al. (2020) documented an inverse relationship between metabolic rate and lifespan in the same fly species (also see Menail et al., 2022). In our case, *A*. *ludens* females are known to invest significantly in costly ovary development and oogenesis (Aluja et al., 2001) which added to the activation to heat stress response mechanisms, may explain why they lived for shorter periods than males.

In conclusion, among the most interesting findings the unexpected cold hardiness of *A*. *obliqua* stands out, a species found in very hot environments. But this species belongs to the same *fraterculus* species group where *A*. *ludens* is placed, the other species that proved better adapted to lower temperatures. In contrast, representatives of the *serpentina* species group withstood better extreme hot temperatures, which means that they may be better adapted to the rising temperatures related to global warming. In the field, we found significant differences in *A*. *ludens* between female and male survival, likely related to the metabolic cost of dealing with highly variable environmental conditions (differences of almost 30°C between the hottest and coldest temperatures were recorded) and ovary development/egg production. Our findings, of ecological nature, demand an in depth look into the physiological/molecular mechanisms behind the patterns observed, although we can rely on the vast literature on *Drosophila* and the few studies on tephritid flies we cite here to infer the types of metabolic processes at play as thermal responses trigger highly conserved metabolic routes in insects (González-Tokman et al., 2020). An area that definitively deserves closer attention is the possible role that bacteria play in aiding tephritid flies in dealing with thermal stress as recently Ayyasamy et al. (2021) identified various bacteria (e.g., *Acinetobacter*, *Brevibacillus*, *Bacillus*, *Enterobacter*, *Enterococcus*, *Pseudomonas* and *Staphylococcus*) associated with resistance to thermal stress in the tefritid fly *B*. *dorsalis*. Another topic worthy of investigation is the one related to the effect of temperature stress and insect immunity (Wojda, 2017), as pestiferous flies are mass reared and sometimes larvae experience high temperatures in their rearing medium caused by metabolic heat which could compromise their immune system and render them more prone to infections by pathogenic bacteria and fungi.

## Data Availability

The raw data supporting the conclusions of this article are available on request to the corresponding authors.
