# Supplementary material for: Coping with global warming: Adult thermal thresholds in four pestiferous Anastrepha species determined under experimental laboratory conditions and development/survival times of immatures and adults under natural field conditions
Source: Front Physiol. 2022 Oct 11;13:991923. doi: 10.3389/fphys.2022.991923 (PMC9593313; doi:10.3389/fphys.2022.991923)
Supplement: Supplementary file 1 [file DataSheet1.docx]

**Supplementary Material**

**Running tittle:** Thermal thresholds in four *Anastrepha* species

Coping with global warming: Adult thermal thresholds in four pestiferous *Anastrepha* species determined under experimental laboratory conditions and development/survival times of immatures and adults under natural field condition

Larissa Guillén^1*^, Carlos Pascacio-Villafán^1^, Ixchel Osorio-Paz^1^, Rafael Ortega-Casas^1^, Erick Enciso-Ortíz^1^, Alma Altuzar-Molina^1^, Olinda Velázquez^1^ & Martín Aluja^1*^

^1^ Red de Manejo Biorracional de Plagas y Vectores, Clúster Científico y Tecnológico BioMimic^®^, Instituto de Ecología, A.C. – INECOL, Carretera Antigua a Coatepec No. 351, Congregación El Haya, 91070 Xalapa, Veracruz, Mexico

* **Authors for correspondence**: [larissa.guillen@inecol.mx](mailto:larissa.guillen@inecol.mx);
 [martin.aluja@inecol.mx](mailto:martin.aluja@inecol.mx)

**Supplementary Tables**

**Table S1** ANOVA and summary statistics of the sixth order polynomial model fitted to data on female life expectancy as a function of temperature, fly species and their interactions.

| **Response** | **1** | **Tiempo_Promedio_De_Vida_De_Las_Hembras_Por_Jaula (D)** |  |
| --- | --- | --- | --- |
| **Transform:** | **Natural Log** | **Constant:** | **0.000000** |

| **ANOVA for Response Surface Sixth model** | | | | | | |
| --- | --- | --- | --- | --- | --- | --- |
| **Analysis of variance table [Classical sum of squares - Type II]** | | | | | | |
|  | **Sum of** |  | **Mean** | **F** | **p-value** |  |
| **Source** | **Squares** | **df** | **Square** | **Value** | **Prob > F** |  |
| Model | 404.27 | 24 | 16.84 | 195.69 | < 0.0001 |  |
| *A-Temperature* | *119.25* | *1* | *119.25* | *1385.35* | *< 0.0001* |  |
| *B-FlySpecies* | *11.04* | *3* | *3.68* | *42.75* | *< 0.0001* |  |
| *AB* | *17.80* | *3* | *5.93* | *68.92* | *< 0.0001* |  |
| *A^2^* | *227.93* | *1* | *227.93* | *2647.95* | *< 0.0001* |  |
| *A^2^B* | *5.15* | *3* | *1.72* | *19.93* | *< 0.0001* |  |
| *A^3^* | *7.76* | *1* | *7.76* | *90.10* | *< 0.0001* |  |
| *A^3^B* | *3.52* | *3* | *1.17* | *13.64* | *< 0.0001* |  |
| *A^4^* | *1.11* | *1* | *1.11* | *12.92* | *0.0005* |  |
| *A^4^B* | *0.72* | *3* | *0.24* | *2.79* | *0.0438* |  |
| *A^5^* | *2.40* | *1* | *2.40* | *27.92* | *< 0.0001* |  |
| *A^5^B* | *3.75* | *3* | *1.25* | *14.51* | *< 0.0001* |  |
| *A^6^* | *3.85* | *1* | *3.85* | *44.74* | *< 0.0001* |  |
| Residual | 9.90 | 115 | 0.086 |  |  |  |
| *Lack of Fit* | *0.11* | *3* | *0.035* | *0.40* | *0.7514* |  |
| *Pure Error* | *9.79* | *112* | *0.087* |  |  |  |
| Cor Total | 414.17 | 139 |  |  |  |  |

| Std. Dev. | 0.29 |  | R-Squared | 0.9761 |
| --- | --- | --- | --- | --- |
| Mean | 2.63 |  | Adj R-Squared | 0.9711 |
| C.V. % | 11.17 |  | Pred R-Squared | 0.9645 |
| PRESS | 14.71 |  | Adeq Precision | 42.692 |
| -2 Log Likelihood | 26.41 |  | BIC | 149.95 |
|  |  |  | AICc | 87.82 |

|  | **Coefficient** |  | **Standard** | **95% CI** | **95% CI** |  |
| --- | --- | --- | --- | --- | --- | --- |
| **Term** | **Estimate** | **df** | **Error** | **Low** | **High** | **VIF** |
| Intercept | 3.78 | 1 | 0.10 | 3.58 | 3.98 |  |
| A-Temperature | -0.57 | 1 | 0.22 | -1.01 | -0.13 | 40.17 |
| B[1] | 0.037 | 1 | 0.098 | -0.16 | 0.23 |  |
| B[2] | -0.017 | 1 | 0.098 | -0.21 | 0.18 |  |
| B[3] | -0.038 | 1 | 0.098 | -0.23 | 0.16 |  |
| AB[1] | -0.64 | 1 | 0.34 | -1.31 | 0.032 |  |
| AB[2] | 1.91 | 1 | 0.34 | 1.24 | 2.58 |  |
| AB[3] | -0.40 | 1 | 0.34 | -1.06 | 0.27 |  |
| A^2^ | 4.61 | 1 | 1.36 | 1.91 | 7.31 | 410.14 |
| A^2^B[1] | 1.28 | 1 | 0.48 | 0.33 | 2.24 |  |
| A^2^B[2] | -0.46 | 1 | 0.48 | -1.42 | 0.50 |  |
| A^2^B[3] | -7.618E-003 | 1 | 0.48 | -0.97 | 0.95 |  |
| A^3^ | -3.74 | 1 | 0.69 | -5.11 | -2.38 | 263.10 |
| A^3^B[1] | -2.22 | 1 | 1.12 | -4.44 | -6.467E-003 |  |
| A^3^B[2] | -5.86 | 1 | 1.12 | -8.07 | -3.64 |  |
| A^3^B[3] | 2.53 | 1 | 1.12 | 0.32 | 4.75 |  |
| A^4^ | -21.39 | 1 | 3.33 | -27.99 | -14.79 | 2994.91 |
| A^4^B[1] | -0.82 | 1 | 0.44 | -1.70 | 0.052 |  |
| A^4^B[2] | 0.89 | 1 | 0.44 | 0.017 | 1.76 |  |
| A^4^B[3] | -0.61 | 1 | 0.44 | -1.48 | 0.26 |  |
| A^5^ | 3.45 | 1 | 0.50 | 2.46 | 4.44 | 122.49 |
| A^5^B[1] | 2.33 | 1 | 0.83 | 0.69 | 3.98 |  |
| A^5^B[2] | 3.60 | 1 | 0.83 | 1.95 | 5.25 |  |
| A^5^B[3] | -1.51 | 1 | 0.83 | -3.16 | 0.14 |  |
| A^6^ | 13.89 | 1 | 2.08 | 9.78 | 18.01 | 1263.06 |

| **Final Equation in Terms of Coded Factors:** | |
| --- | --- |
| Ln(Tiempo_Promedio_De_Vida_De_Las_Hembras_Por_Jaula (D) ) | = |
| +3.78 |  |
| -0.57 | * A |
| +0.037 | * B[1] |
| -0.017 | * B[2] |
| -0.038 | * B[3] |
| -0.64 | * AB[1] |
| +1.91 | * AB[2] |
| -0.40 | * AB[3] |
| +4.61 | * A^2^ |
| +1.28 | * A^2^B[1] |
| -0.46 | * A^2^B[2] |
| -7.618E-003 | * A^2^B[3] |
| -3.74 | * A^3^ |
| -2.22 | * A^3^B[1] |
| -5.86 | * A^3^B[2] |
| +2.53 | * A^3^B[3] |
| -21.39 | * A^4^ |
| -0.82 | * A^4^B[1] |
| +0.89 | * A^4^B[2] |
| -0.61 | * A^4^B[3] |
| +3.45 | * A^5^ |
| +2.33 | * A^5^B[1] |
| +3.60 | * A^5^B[2] |
| -1.51 | * A^5^B[3] |
| +13.89 | * A^6^ |

| **Final Equation in Terms of Actual Factors:** | |
| --- | --- |
| FlySpecies | A. ludens |
| Ln(Tiempo_Promedio_De_Vida_De_Las_Hembras_Por_Jaula (D) ) | = |
| +7.32308 |  |
| -2.71077 | * Temperature |
| +0.53983 | * Temperature^2^ |
| -0.043415 | * Temperature^3^ |
| +1.67064E-003 | * Temperature^4^ |
| -3.07580E-005 | * Temperature^5^ |
| +2.17097E-007 | * Temperature^6^ |
| FlySpecies | A. obliqua |
| Ln(Tiempo_Promedio_De_Vida_De_Las_Hembras_Por_Jaula (D) ) | = |
| +8.78469 |  |
| -3.11543 | * Temperature |
| +0.54802 | * Temperature^2^ |
| -0.042473 | * Temperature^3^ |
| +1.63203E-003 | * Temperature^4^ |
| -3.03636E-005 | * Temperature^5^ |
| +2.17097E-007 | * Temperature^6^ |
| FlySpecies | A. serpentina |
| Ln(Tiempo_Promedio_De_Vida_De_Las_Hembras_Por_Jaula (D) ) | = |
| +7.87621 |  |
| -3.84964 | * Temperature |
| +0.68454 | * Temperature^2^ |
| -0.050455 | * Temperature^3^ |
| +1.82203E-003 | * Temperature^4^ |
| -3.19587E-005 | * Temperature^5^ |
| +2.17097E-007 | * Temperature^6^ |
| FlySpecies | A. striata |
| Ln(Tiempo_Promedio_De_Vida_De_Las_Hembras_Por_Jaula (D) ) | = |
| +13.11138 |  |
| -5.29799 | * Temperature |
| +0.82376 | * Temperature^2^ |
| -0.056497 | * Temperature^3^ |
| +1.94318E-003 | * Temperature^4^ |
| -3.28701E-005 | * Temperature^5^ |
| +2.17097E-007 | * Temperature^6^ |

**Table S2** ANOVA and summary statistics of the sixth order polynomial model fitted to data on male life expectancy as a function of temperature, fly species and their interactions.

| **Response** | **2** | **Tiempo_Promedio_De_Vida_De_Los_Machos_Por_Jaula** |  |
| --- | --- | --- | --- |
| **Transform:** | **Natural Log** | **Constant:** | **0.000000** |

| **ANOVA for Response Surface Sixth model** | | | | | | |
| --- | --- | --- | --- | --- | --- | --- |
| **Analysis of variance table [Classical sum of squares - Type II]** | | | | | | |
|  | **Sum of** |  | **Mean** | **F** | **p-value** |  |
| **Source** | **Squares** | **df** | **Square** | **Value** | **Prob > F** |  |
| Model | 396.50 | 24 | 16.52 | 156.21 | < 0.0001 |  |
| *A-Temperature* | *103.60* | *1* | *103.60* | *979.59* | *< 0.0001* |  |
| *B-FlySpecies* | *11.51* | *3* | *3.84* | *36.26* | *< 0.0001* |  |
| *AB* | *14.30* | *3* | *4.77* | *45.08* | *< 0.0001* |  |
| *A^2^* | *241.32* | *1* | *241.32* | *2281.81* | *< 0.0001* |  |
| *A^2^B* | *4.51* | *3* | *1.50* | *14.20* | *< 0.0001* |  |
| *A^3^* | *6.34* | *1* | *6.34* | *59.99* | *< 0.0001* |  |
| *A^3^B* | *4.16* | *3* | *1.39* | *13.12* | *< 0.0001* |  |
| *A^4^* | *2.70* | *1* | *2.70* | *25.53* | *< 0.0001* |  |
| *A^4^B* | *1.36* | *3* | *0.45* | *4.29* | *0.0066* |  |
| *A^5^* | *3.57* | *1* | *3.57* | *33.79* | *< 0.0001* |  |
| *A^5^B* | *1.84* | *3* | *0.61* | *5.81* | *0.0010* |  |
| *A^6^* | *1.28* | *1* | *1.28* | *12.14* | *0.0007* |  |
| Residual | 12.16 | 115 | 0.11 |  |  |  |
| *Lack of Fit* | *0.15* | *3* | *0.050* | *0.47* | *0.7033* |  |
| *Pure Error* | *12.01* | *112* | *0.11* |  |  |  |
| Cor Total | 408.66 | 139 |  |  |  |  |

| Std. Dev. | 0.33 |  | R-Squared | 0.9702 |
| --- | --- | --- | --- | --- |
| Mean | 2.55 |  | Adj R-Squared | 0.9640 |
| C.V. % | 12.74 |  | Pred R-Squared | 0.9558 |
| PRESS | 18.06 |  | Adeq Precision | 36.044 |
| -2 Log Likelihood | 55.24 |  | BIC | 178.78 |
|  |  |  | AICc | 116.64 |

|  | **Coefficient** |  | **Standard** | **95% CI** | **95% CI** |  |
| --- | --- | --- | --- | --- | --- | --- |
| **Term** | **Estimate** | **df** | **Error** | **Low** | **High** | **VIF** |
| Intercept | 4.08 | 1 | 0.11 | 3.86 | 4.30 |  |
| A-Temperature | -0.53 | 1 | 0.25 | -1.03 | -0.044 | 40.17 |
| B[1] | 0.18 | 1 | 0.11 | -0.034 | 0.40 |  |
| B[2] | -8.052E-003 | 1 | 0.11 | -0.22 | 0.21 |  |
| B[3] | -0.16 | 1 | 0.11 | -0.37 | 0.057 |  |
| AB[1] | -0.23 | 1 | 0.37 | -0.97 | 0.51 |  |
| AB[2] | 1.54 | 1 | 0.37 | 0.80 | 2.28 |  |
| AB[3] | -0.50 | 1 | 0.37 | -1.24 | 0.24 |  |
| A^2^ | 0.21 | 1 | 1.51 | -2.78 | 3.20 | 410.14 |
| A^2^B[1] | 0.82 | 1 | 0.54 | -0.25 | 1.88 |  |
| A^2^B[2] | -1.25 | 1 | 0.54 | -2.31 | -0.18 |  |
| A^2^B[3] | -0.017 | 1 | 0.54 | -1.08 | 1.05 |  |
| A^3^ | -3.87 | 1 | 0.77 | -5.39 | -2.35 | 263.10 |
| A^3^B[1] | -3.13 | 1 | 1.24 | -5.59 | -0.67 |  |
| A^3^B[2] | -3.38 | 1 | 1.24 | -5.83 | -0.92 |  |
| A^3^B[3] | 2.84 | 1 | 1.24 | 0.39 | 5.30 |  |
| A^4^ | -11.51 | 1 | 3.69 | -18.82 | -4.19 | 2994.91 |
| A^4^B[1] | -0.49 | 1 | 0.49 | -1.46 | 0.48 |  |
| A^4^B[2] | 1.68 | 1 | 0.49 | 0.71 | 2.65 |  |
| A^4^B[3] | -0.40 | 1 | 0.49 | -1.37 | 0.57 |  |
| A^5^ | 3.62 | 1 | 0.55 | 2.52 | 4.72 | 122.49 |
| A^5^B[1] | 2.83 | 1 | 0.92 | 1.00 | 4.66 |  |
| A^5^B[2] | 1.49 | 1 | 0.92 | -0.34 | 3.32 |  |
| A^5^B[3] | -1.79 | 1 | 0.92 | -3.62 | 0.038 |  |
| A^6^ | 8.02 | 1 | 2.30 | 3.46 | 12.58 | 1263.06 |

| **Final Equation in Terms of Coded Factors:** | |
| --- | --- |
| Ln(Tiempo_Promedio_De_Vida_De_Los_Machos_Por_Jaula) | = |
| +4.08 |  |
| -0.53 | * A |
| +0.18 | * B[1] |
| -8.052E-003 | * B[2] |
| -0.16 | * B[3] |
| -0.23 | * AB[1] |
| +1.54 | * AB[2] |
| -0.50 | * AB[3] |
| +0.21 | * A^2^ |
| +0.82 | * A^2^B[1] |
| -1.25 | * A^2^B[2] |
| -0.017 | * A^2^B[3] |
| -3.87 | * A^3^ |
| -3.13 | * A^3^B[1] |
| -3.38 | * A^3^B[2] |
| +2.84 | * A^3^B[3] |
| -11.51 | * A^4^ |
| -0.49 | * A^4^B[1] |
| +1.68 | * A^4^B[2] |
| -0.40 | * A^4^B[3] |
| +3.62 | * A^5^ |
| +2.83 | * A^5^B[1] |
| +1.49 | * A^5^B[2] |
| -1.79 | * A^5^B[3] |
| +8.02 | * A^6^ |

| **Final Equation in Terms of Actual Factors:** | |
| --- | --- |
| FlySpecies | A. ludens |
| Ln(Tiempo_Promedio_De_Vida_De_Los_Machos_Por_Jaula) | = |
| +2.12065 |  |
| -0.52886 | * Temperature |
| +0.20660 | * Temperature^2^ |
| -0.019955 | * Temperature^3^ |
| +8.48324E-004 | * Temperature^4^ |
| -1.67882E-005 | * Temperature^5^ |
| +1.25356E-007 | * Temperature^6^ |
| FlySpecies | A. obliqua |
| Ln(Tiempo_Promedio_De_Vida_De_Los_Machos_Por_Jaula) | = |
| +6.36768 |  |
| -1.90638 | * Temperature |
| +0.32008 | * Temperature^2^ |
| -0.023960 | * Temperature^3^ |
| +9.14255E-004 | * Temperature^4^ |
| -1.72072E-005 | * Temperature^5^ |
| +1.25356E-007 | * Temperature^6^ |
| FlySpecies | A. serpentina |
| Ln(Tiempo_Promedio_De_Vida_De_Los_Machos_Por_Jaula) | = |
| +3.46092 |  |
| -1.89190 | * Temperature |
| +0.37611 | * Temperature^2^ |
| -0.028284 | * Temperature^3^ |
| +1.02927E-003 | * Temperature^4^ |
| -1.82312E-005 | * Temperature^5^ |
| +1.25356E-007 | * Temperature^6^ |
| FlySpecies | A. striata |
| Ln(Tiempo_Promedio_De_Vida_De_Los_Machos_Por_Jaula) | = |
| +4.43547 |  |
| -2.07277 | * Temperature |
| +0.39650 | * Temperature^2^ |
| -0.029377 | * Temperature^3^ |
| +1.05558E-003 | * Temperature^4^ |
| -1.84608E-005 | * Temperature^5^ |
| +1.25356E-007 | * Temperature^6^ |

**Table S3** ANOVA and summary statistics of the cubic model fitted to data on daily egg production per female fly as a function of temperature, fly species and their interactions.

| **Response** | **4** | **HuvosPorDorHmebra** |  |
| --- | --- | --- | --- |
| **Transform:** | **Natural Log** | **Constant:** | **0.050988** |

| **ANOVA for Response Surface Cubic model** | | | | | | |
| --- | --- | --- | --- | --- | --- | --- |
| **Analysis of variance table [Classical sum of squares - Type II]** | | | | | | |
|  | **Sum of** |  | **Mean** | **F** | **p-value** |  |
| **Source** | **Squares** | **df** | **Square** | **Value** | **Prob > F** |  |
| Model | 454.29 | 12 | 37.86 | 45.08 | < 0.0001 |  |
| *A-Temperature* | *253.41* | *1* | *253.41* | *301.76* | *< 0.0001* |  |
| *B-FlySpecies* | *73.08* | *3* | *24.36* | *29.01* | *< 0.0001* |  |
| *AB* | *29.85* | *3* | *9.95* | *11.85* | *< 0.0001* |  |
| *A^2^* | *27.00* | *1* | *27.00* | *32.15* | *< 0.0001* |  |
| *A^2^B* | *12.85* | *3* | *4.28* | *5.10* | *0.0028* |  |
| *A^3^* | *25.82* | *1* | *25.82* | *30.74* | *< 0.0001* |  |
| Residual | 68.86 | 82 | 0.84 |  |  |  |
| *Lack of Fit* | *60.52* | *7* | *8.65* | *77.74* | *< 0.0001* |  |
| *Pure Error* | *8.34* | *75* | *0.11* |  |  |  |
| Cor Total | 523.15 | 94 |  |  |  |  |

| Std. Dev. | 0.92 |  | R-Squared | 0.8684 |
| --- | --- | --- | --- | --- |
| Mean | -0.86 |  | Adj R-Squared | 0.8491 |
| C.V. % | 106.94 |  | Pred R-Squared | 0.1906 |
| PRESS | 423.45 |  | Adeq Precision | 25.330 |
| -2 Log Likelihood | 239.03 |  | BIC | 298.23 |
|  |  |  | AICc | 269.52 |

|  | **Coefficient** |  | **Standard** | **95% CI** | **95% CI** |  |
| --- | --- | --- | --- | --- | --- | --- |
| **Term** | **Estimate** | **df** | **Error** | **Low** | **High** | **VIF** |
| Intercept | 1.78 | 1 | 0.18 | 1.41 | 2.14 |  |
| A-Temperature | 2.46 | 1 | 0.43 | 1.60 | 3.33 | 4.03 |
| B[1] | 2.29 | 1 | 0.24 | 1.82 | 2.77 |  |
| B[2] | -1.03 | 1 | 0.24 | -1.51 | -0.55 |  |
| B[3] | 0.47 | 1 | 0.27 | -0.059 | 1.00 |  |
| AB[1] | -0.057 | 1 | 0.79 | -1.63 | 1.51 |  |
| AB[2] | -0.32 | 1 | 0.79 | -1.89 | 1.25 |  |
| AB[3] | -0.022 | 1 | 0.84 | -1.70 | 1.65 |  |
| A^2^ | -10.11 | 1 | 1.35 | -12.78 | -7.43 | 22.34 |
| A^2^B[1] | -2.19 | 1 | 1.03 | -4.24 | -0.14 |  |
| A^2^B[2] | 1.18 | 1 | 1.03 | -0.87 | 3.23 |  |
| A^2^B[3] | -2.01 | 1 | 1.46 | -4.91 | 0.89 |  |
| A^3^ | -7.09 | 1 | 1.28 | -9.63 | -4.54 | 22.83 |

| **Final Equation in Terms of Coded Factors:** | |
| --- | --- |
| Ln(HuvosPorDorHmebra + 0.05) | = |
| +1.78 |  |
| +2.46 | * A |
| +2.29 | * B[1] |
| -1.03 | * B[2] |
| +0.47 | * B[3] |
| -0.057 | * AB[1] |
| -0.32 | * AB[2] |
| -0.022 | * AB[3] |
| -10.11 | * A^2^ |
| -2.19 | * A^2^B[1] |
| +1.18 | * A^2^B[2] |
| -2.01 | * A^2^B[3] |
| -7.09 | * A^3^ |

| **Final Equation in Terms of Actual Factors:** | |
| --- | --- |
| FlySpecies | A. ludens |
| Ln(HuvosPorDorHmebra + 0.05) | = |
| -4.30517 |  |
| -4.00073E-003 | * Temperature |
| +0.035708 | * Temperature^2^ |
| -8.85880E-004 | * Temperature^3^ |
| FlySpecies | A. obliqua |
| Ln(HuvosPorDorHmebra + 0.05) | = |
| -2.04249 |  |
| -0.43787 | * Temperature |
| +0.044127 | * Temperature^2^ |
| -8.85880E-004 | * Temperature^3^ |
| FlySpecies | A. serpentina |
| Ln(HuvosPorDorHmebra + 0.05) | = |
| -5.88988 |  |
| -0.024640 | * Temperature |
| +0.036156 | * Temperature^2^ |
| -8.85880E-004 | * Temperature^3^ |
| FlySpecies | A. striata |
| Ln(HuvosPorDorHmebra + 0.05) | = |
| -0.77365 |  |
| -0.63149 | * Temperature |
| +0.048710 | * Temperature^2^ |
| -8.85880E-004 | * Temperature^3^ |

**Table S4** ANOVA and summary statistics of the quadratic model fitted to data on egg hatch as a function of temperature, fly species and their interactions.

| **Response** | **5** | **Porcentaje_Promedio_De_EclosiNBon_Por_Jaula** |  |  |  |
| --- | --- | --- | --- | --- | --- |
| **Transform:** | **Logit** | **Lower bound:** | **-1.000000E-002** | **Upper bound:** | **1.010000** |

| **ANOVA for Response Surface Quadratic model** | | | | | | |
| --- | --- | --- | --- | --- | --- | --- |
| **Analysis of variance table [Classical sum of squares - Type II]** | | | | | | |
|  | **Sum of** |  | **Mean** | **F** | **p-value** |  |
| **Source** | **Squares** | **df** | **Square** | **Value** | **Prob > F** |  |
| Model | 97.73 | 8 | 12.22 | 17.06 | < 0.0001 |  |
| *A-Temperature* | *25.48* | *1* | *25.48* | *35.58* | *< 0.0001* |  |
| *B-FlySpecies* | *50.04* | *3* | *16.68* | *23.30* | *< 0.0001* |  |
| *AB* | *6.22* | *3* | *2.07* | *2.90* | *0.0465* |  |
| *A^2^* | *37.02* | *1* | *37.02* | *51.71* | *< 0.0001* |  |
| Residual | 29.35 | 41 | 0.72 |  |  |  |
| *Lack of Fit* | *8.41* | *2* | *4.20* | *7.82* | *0.0014* |  |
| *Pure Error* | *20.95* | *39* | *0.54* |  |  |  |
| Cor Total | 127.08 | 49 |  |  |  |  |

| Std. Dev. | 0.85 |  | R-Squared | 0.7690 |
| --- | --- | --- | --- | --- |
| Mean | -2.79 |  | Adj R-Squared | 0.7240 |
| C.V. % | 30.29 |  | Pred R-Squared | 0.6599 |
| PRESS | 43.23 |  | Adeq Precision | 12.676 |
| -2 Log Likelihood | 115.26 |  | BIC | 150.47 |
|  |  |  | AICc | 137.76 |

|  | **Coefficient** |  | **Standard** | **95% CI** | **95% CI** |  |
| --- | --- | --- | --- | --- | --- | --- |
| **Term** | **Estimate** | **df** | **Error** | **Low** | **High** | **VIF** |
| Intercept | -1.24 | 1 | 0.23 | -1.71 | -0.78 |  |
| A-Temperature | 0.013 | 1 | 0.54 | -1.07 | 1.09 | 1.80 |
| B[1] | 1.60 | 1 | 0.22 | 1.16 | 2.04 |  |
| B[2] | 0.26 | 1 | 0.22 | -0.20 | 0.71 |  |
| B[3] | -0.41 | 1 | 0.22 | -0.85 | 0.031 |  |
| AB[1] | 0.12 | 1 | 0.69 | -1.28 | 1.51 |  |
| AB[2] | -0.24 | 1 | 0.78 | -1.82 | 1.34 |  |
| AB[3] | -1.86 | 1 | 0.70 | -3.27 | -0.45 |  |
| A^2^ | -17.53 | 1 | 2.44 | -22.45 | -12.60 | 2.33 |

| **Final Equation in Terms of Coded Factors:** | |
| --- | --- |
| Logit(Porcentaje_Promedio_De_EclosiNBon_Por_Jaula ) | = Ln[(Porcentaje_Promedio_De_EclosiNBon_Por_Jaula + 0.01)/(1.01 - Porcentaje_Promedio_De_EclosiNBon_Por_Jaula )] = |
| -1.24 |  |
| +0.013 | * A |
| +1.60 | * B[1] |
| +0.26 | * B[2] |
| -0.41 | * B[3] |
| +0.12 | * AB[1] |
| -0.24 | * AB[2] |
| -1.86 | * AB[3] |
| -17.53 | * A^2^ |

| **Final Equation in Terms of Actual Factors:** | |
| --- | --- |
| FlySpecies | A. ludens |
| Logit(Porcentaje_Promedio_De_EclosiNBon_Por_Jaula ) | = Ln[(Porcentaje_Promedio_De_EclosiNBon_Por_Jaula + 0.01)/(1.01 - Porcentaje_Promedio_De_EclosiNBon_Por_Jaula )] = |
| -27.19027 |  |
| +2.19725 | * Temperature |
| -0.043814 | * Temperature^2^ |
| FlySpecies | A. obliqua |
| Logit(Porcentaje_Promedio_De_EclosiNBon_Por_Jaula ) | = Ln[(Porcentaje_Promedio_De_EclosiNBon_Por_Jaula + 0.01)/(1.01 - Porcentaje_Promedio_De_EclosiNBon_Por_Jaula )] = |
| -28.08884 |  |
| +2.17941 | * Temperature |
| -0.043814 | * Temperature^2^ |
| FlySpecies | A. serpentina |
| Logit(Porcentaje_Promedio_De_EclosiNBon_Por_Jaula ) | = Ln[(Porcentaje_Promedio_De_EclosiNBon_Por_Jaula + 0.01)/(1.01 - Porcentaje_Promedio_De_EclosiNBon_Por_Jaula )] = |
| -26.72941 |  |
| +2.09840 | * Temperature |
| -0.043814 | * Temperature^2^ |
| FlySpecies | A. striata |
| Logit(Porcentaje_Promedio_De_EclosiNBon_Por_Jaula ) | = Ln[(Porcentaje_Promedio_De_EclosiNBon_Por_Jaula + 0.01)/(1.01 - Porcentaje_Promedio_De_EclosiNBon_Por_Jaula )] = |
| -32.56398 |  |
| +2.29038 | * Temperature |
| -0.043814 | * Temperature^2^ |

**Table S5** ANOVA and summary statistics of the two factor interaction model model fitted to data on egg development time as a function of temperature, fly species and their interactions.

| **Response** | **6** | **Tiempo_Promedio_De_Eclosion_Por_Jaula** |
| --- | --- | --- |

| **ANOVA for Response Surface 2FI model** | | | | | | |
| --- | --- | --- | --- | --- | --- | --- |
| **Analysis of variance table [Classical sum of squares - Type II]** | | | | | | |
|  | **Sum of** |  | **Mean** | **F** | **p-value** |  |
| **Source** | **Squares** | **df** | **Square** | **Value** | **Prob > F** |  |
| Model | 87.30 | 7 | 12.47 | 33.94 | < 0.0001 |  |
| *A-Temperature* | *69.02* | *1* | *69.02* | *187.84* | *< 0.0001* |  |
| *B-FlySpecies* | *5.99* | *3* | *2.00* | *5.43* | *0.0043* |  |
| *AB* | *10.67* | *3* | *3.56* | *9.68* | *0.0001* |  |
| Pure Error | 10.66 | 29 | 0.37 |  |  |  |
| Cor Total | 97.95 | 36 |  |  |  |  |

| Std. Dev. | 0.61 |  | R-Squared | 0.8912 |
| --- | --- | --- | --- | --- |
| Mean | 5.53 |  | Adj R-Squared | 0.8649 |
| C.V. % | 10.97 |  | Pred R-Squared | 0.7854 |
| PRESS | 21.03 |  | Adeq Precision | 13.476 |
| -2 Log Likelihood | 58.95 |  | BIC | 87.83 |
|  |  |  | AICc | 80.09 |

|  | **Coefficient** |  | **Standard** | **95% CI** | **95% CI** |  |
| --- | --- | --- | --- | --- | --- | --- |
| **Term** | **Estimate** | **df** | **Error** | **Low** | **High** | **VIF** |
| Intercept | 5.56 | 1 | 0.10 | 5.36 | 5.77 |  |
| A-Temperature | -5.16 | 1 | 0.40 | -5.99 | -4.33 | 1.03 |
| B[1] | 0.14 | 1 | 0.17 | -0.21 | 0.48 |  |
| B[2] | -0.68 | 1 | 0.17 | -1.03 | -0.34 |  |
| B[3] | -0.028 | 1 | 0.17 | -0.37 | 0.32 |  |
| AB[1] | -1.87 | 1 | 0.68 | -3.25 | -0.48 |  |
| AB[2] | 0.27 | 1 | 0.68 | -1.12 | 1.65 |  |
| AB[3] | -2.11 | 1 | 0.68 | -3.49 | -0.73 |  |

| **Final Equation in Terms of Coded Factors:** | |
| --- | --- |
| Tiempo_Promedio_De_Eclosion_Por_Jaula | = |
| +5.56 |  |
| -5.16 | * A |
| +0.14 | * B[1] |
| -0.68 | * B[2] |
| -0.028 | * B[3] |
| -1.87 | * AB[1] |
| +0.27 | * AB[2] |
| -2.11 | * AB[3] |

| **Final Equation in Terms of Actual Factors:** | |
| --- | --- |
| FlySpecies | A. ludens |
| Tiempo_Promedio_De_Eclosion_Por_Jaula | = |
| +14.47785 |  |
| -0.35116 | * Temperature |
| FlySpecies | A. obliqua |
| Tiempo_Promedio_De_Eclosion_Por_Jaula | = |
| +10.99174 |  |
| -0.24452 | * Temperature |
| FlySpecies | A. serpentina |
| Tiempo_Promedio_De_Eclosion_Por_Jaula | = |
| +14.62063 |  |
| -0.36342 | * Temperature |
| FlySpecies | A. striata |
| Tiempo_Promedio_De_Eclosion_Por_Jaula | = |
| +7.95245 |  |
| -0.072449 | * Temperature |
